# Aeolian accumulation rate within the kettle holes on Skeiðarársandur (S Iceland) under climate warming conditions

**DOI:** 10.1038/s41598-025-07346-2

**Published:** 2025-07-02

**Authors:** Joanna Ewa Szafraniec

**Affiliations:** Faculty of Natural Sciences, Institute of Earth Sciences, University of Silesia in Katowice, Sosnowiec, 41-200 Poland

**Keywords:** Skeiðarársandur outwash plain, Kettle holes, Aeolian accumulation, Glacial lake-outburst flood (jökulhlaup), Climate warming, Geomorphology, Sedimentology, Environmental impact, Climate change

## Abstract

Like other higher latitudes, Iceland is experiencing climate warming, leading to glacier retreat and the formation of new plume areas of aeolian material – vast deserts, including the Skeiðarársandur outwash plain. The main objective was determining the annual rate of aeolian accumulation within glacial flood-origin sandur kettle holes as important sediment traps. The analysis was based on data collected in 2021/2022–2023/2024, at an older level, in depressions after 1892 and 1934/1938 glacial lake-outburst floods, and at a younger level, after the catastrophic jökulhlaup of 1996. Meteorological data, digital elevation models, satellite scenes and photogrammetric data were also used to examine the role of wind, morphometry of kettle holes, and plant coverage in the aeolian accumulation rate and to validate the monitoring results. Analysis showed that the material collected in the kettle holes was of local origin. On the younger level, without vegetation or an initial succession, particle transport occured mostly via saltation (63–75%), and the aeolian aacumulation rate averaged 5,000–7,400 g m^–2^ yr^–1^ (50% between 3,600 and 13,400 g m^–2^ yr^–1^). It is several times higher than on the flat sandur surface and 6–44 times higher than on the older level, completely covered with vegetation, and more distant from plume areas. Mineral particles here were finer and transported mainly in suspension (50–85%). Identifying aeolian facies and particle characteristics within the analogous Pleistocene sandur kettle holes of the glacial lake-outburst flood-origin in the European Lowland may indicate the time of their formation caused by climate warming, the Scandinavian Ice Sheet decay, and the transformation of ecosystems from tundra to forest.

**Supplementary Information:**

The online version contains supplementary material available at 10.1038/s41598-025-07346-2.

## Introduction

Aeolian accumulation (the difference between deposition and erosion^1^) reaches high values ​​in deserts, loess zones, sea coasts and peri-desert mountains^2,3^. Such desert areas also include contemporary and Pleistocene glaciers and ice sheet marginal zones. Regardless of the period of deglaciation that occurs due to climate warming, environmental changes usually propagate very quickly (e.g.^4–9^), which results, among other things, in the expansion of ice-free areas (e.g.^10^). In the case of mountain glaciation, the volume of glaciers decreases not only due to their frontal retreat but also through the ice surface lowering (e.g.^11^). In this way, the upper parts of slopes in valleys and nunataks emerge from under the ice, where permafrost still dominates. Ice-free rocks and sediments, exposed to intensive cryolithogenesis^12–14^, are the primary source of aeolian material, which, together with strong winds, reaches the periglacial area^15–21^. They form from large-scale atmospheric circulation conditions and high pressure above the ice caps^22^. An example of such a zone was the European Sand Belt with the dune fields^23–25^, formed at the forefield of the Scandinavian Ice Sheet (SIS), south of which the sandy loess and loess were deposited^26^. Material involved in aeolian transport, with sufficiently strong and long-lasting winds, can reach hundreds of kilometres, even overcoming the sea barrier (e.g.^27^).

The most important factors determining the intensity of aeolian processes are the availability of material capable of aeolian transport, the wind regime – its speed and duration, and, partially, the humidity and heat balances^28^. At the boundary of the glacial and proglacial systems, the dynamics of the braided river network play an important role, especially those originating from under the warm-based ice bodies, reflecting the response of glaciers to climate change^29^. Factors inhibiting the development of aeolian processes include vegetation and snow covers, ground freezing, constant ground moisture and surface roughness^28,30^, especially the presence of deflation pavement^31^. However, some research indicates that under certain conditions, even a moist substrate, deflation pavement or grass, due to their elastic properties, may constitute a second surface of bouncing-off for mineral particles^32–34^.

The rate of contemporary aeolian accumulation in high-latitude desert areas is variable. It depends mainly on the distance of the measurement site from plume areas and the length of the observation period^3,29^. In Canada, in the Coastal Mountains of British Columbia, Jones (1982) collected 0.9–6.9 g m^–2^ yr^–1^ of mineral aeolian material in 1981^35^. Owens and Slaymaker (1997), from June 1995 to May 1996, determined the aeolian accumulation rate of mineral particles in three catchments of the same area at 8.48–43.02 g m^–2^ yr^–1^ in non-vegetated terrain, 5.83–7.80 g m^–2^ yr^–1^ in the tundra and 2.90–3.23 g m^–2^ yr^–1^ between trees^36^. In turn, Hugenholtz and Wolfe (2010), based on measurements in the Athabasca River Valley in the Canadian Rocky Mountains conducted during the period 20 May 2007 to 13 May 2009, determined the accumulation rate at 2 to 2,760 g m^–2^ mo^–1^ (24–33,120 g m^–2^ yr^–1^)^37^. In Greenland, in the Kangerlussuaq region, measurements conducted from 18 June to 13 August 2001 by^38^ gave an accumulation value of 3.5 g m^–2^ yr^–1^, and by^39^, from April 2017 to April 2019, an aeolian accumulation rate was 14.6–65.7 g m^–2^ yr^–1^. In western Spitsbergen, measurements in different periods showed about 300–400 g m^–2^ yr^–1^ in the 1957/1958 season^40^, an average of 29 g m^–2^ yr^–1^ in the early 1970s^41^ and an average of 72 g m^–2^ yr^–1^ in the late 1980s, with a maximum value of up to 537 g m^–2^ yr^–1^^28^. During the ablation seasons (June–September) of 2012–2018, in the postglacial Ebba Valley in the central part of Spitsbergen, the deposition rate on the sandur area was calculated at 0.7–7.2 g m^–2^ d^–1^^42^. The authors estimated that the entire Ebba Valley could have been covered with material at a rate of 500–600 g m^–2^ yr^–1^. In the case of Iceland, according to different sources, the typical accumulation rate was estimated by^43^ at 13–26 g m^–2^ yr^–1^ as low to 569–807 g m^–2^ yr^–1^ as very high. The above-mentioned values ​​of mean annual accumulation of > 50 g m^–2^ indicate a local source area of ​​aeolian material localised in the distance < 10 km from the measurement site, and below this value, a source located at a distance of up to 1000 km^3^, including values of 20–50 g m^–2^ yr^–1^ as located closer to the local plume area.

Today, Iceland, like many areas of high latitudes, is experiencing the effects of climate warming^44^. From 1798 to 2007, the mean annual air temperature (MAAT) in the western part of the island increased by 0.7 °C per century^45^. From 1980 to 2019, the rate of temperature increase was 2.6 times faster than the global average rate at c. 0.5 °C per decade^46^. A similar trend was observed in the south of the country, in the forefield of southern Vatnajökull, where summer temperatures increased by an average of 0.15 °C per decade and winter temperatures by 0.3 °C per decade compared to the base period 1981–2000^47^. In the broader context, the continuous trends of increasing winter and spring temperatures in Iceland are significant, as observed during the Common Era (last 2000 years^48^). If these trends continue, the air temperature increase will accompany a precipitation increase of about 5% for every 1 °C warming, mainly rain, especially in winter. In turn, summers, especially June, will become drier^49^. The glaciers cover about 10% of the island, and the ice volume in 2019 was about 3,400 km^3^^50^. However, since 1890, 540 Gt ± 130 Gt of ice has been lost, half of which in 1994/95–2018/19. Glaciers are projected to lose between 43% ±11% and 85% ±7% of their volume by 2100^51^. Other models predict that the ice sheet will halve in the next 100–150 years and disappear within 200 years^47^.

One of the outlet valley glaciers from the Vatnajökull ice cap is Skeiðarárjökull. A vast part of its forefield is an outwash plain, Skeiðarársandur. This desert^43^, with an area of ​​about 1,000 km^2^^52^, is one of the most important Icelandic plume areas of aeolian material of the volcano-fluvial origin^27^ and with the most significant aeolian deposition. The arid area’s accumulation value has been estimated at > 500 g m^–2^ yr^–1^ and for the vegetated area > 250 g m^–2^ yr^–1^^43^.

The sandur is characterised by the diverse spatial extent of plant colonisation observed at different levels, mainly due to its glacial flood genesis^52^. In the early Holocene, these glacial lake-outburst floods (GLOFs), called jökulhlaup in Icelandic, were closely linked to climate warming, the glaciation retreat and the Grímsvötn eruption, as deglaciation and melting favour volcanism^53–56^. It is caused by ice mass thinning over the magma chamber, accelerating isostatic uplift and stress changes in the crust^57^. At least two such episodes are indicated: ca. 11.4–11.2 ka cal and 10.4–9.9 ka cal^58^. Historical data records that GLOFs at Skeiðarársandur have been observed since at least 1201^59^. They are related to the runoff of the Grænalón ice-dammed lake, the breaking of the ice plug in the Grímsvötn volcano subglacial caldera, and/or the volcano eruption, and the glacier surge phenomenon of Skeiðarárjökull. As a result, some parts of the glacier forefield, covered with a series of glaciofluvial sediments, are, in a sense, “reset” regarding vegetation cover.

The end of the Little Ice Age (LIA) cold period increased volcanic intensity and extreme glacial floods (see^59^; Table 2, p. 5–7). The last catastrophic flood occurred in November 1996, when the maximum discharge reached 4 × 10^4^ m^3^ s^–1^^60^. The last 29 years have seen minor GLOFs with maximum discharges of 0.5–4 × 10^3^ m^3^ s^–1^^61–63^ and drainage of the Grænalón lake^59^, sometimes even several times a year, which confirms the increase in ice melting.

A characteristic feature of Skeiðarársandur is the numerous kettle holes. As elements of the ice-marginal land systems (e.g.^64^), they are treated as an indicator of the occurrence of glacial floods (e.g.^65–72^). Aeolian accumulation here (and aeolian-nival), alongside mass movements and water linear erosion, is one of the most important processes contributing to changes in the morphometry of kettle holes in the periods between successive floods (or/and volcanic ejecta), especially at younger levels with a low degree of plant colonisation. Determining the mass balance of sediments in the landforms regarding the dominant processes allows us to understand the rate and nature of changes in morphometry with the passage of time and vegetation encroachment.

Research on the conditions and rate of aeolian accumulation in kettle holes of glacial flood origin has broader implications for reconstructing late Pleistocene/early Holocene palaeoenvironments, e.g. in the European Lowlands. Numerous geomorphological and sedimentological premises and results of hydrological modelling (e.g.^72–87^) indicate that large glacial floods accompanied ice sheet melting. The estimated maximum flow scale in Pomerania (NW Poland), calculated for sandur zones with flood morphological features, could reach 10^5^ m^3^ s^–1^^78^, and according to^72^, even 2 × 10^6^ m^3^ s^–1^ in NE Poland. Some outwash plains of Northern Poland after the Scandinavian glaciation are characterised by the occurrence of kettle holes, for which glacial flood genesis seems probable, such as cluster kettle holes and ice-block obstacle marks from the glacier outburst flood at the end of the Pleistocene^72^. It is, therefore, essential to identify the environmental conditions that have made it possible for these small landforms to survive in the landscape for several thousand years. The oriented kettle holes are associated with the degradation of ice masses formed in proglacial channels^88,89^, remnants of dead ice that prevent tunnel valleys from being filled by outwash deposits^90^ or the degradation of ice blocks deposited during the waning phase of the GLOF in the form of a linear cluster parallel to the jökulhlaup flow^72,91,92^. Van Loon et al. (2012)^88^, Klimek (1997)^93^ and Błaszkiewicz (2011)^94^ indicate that some of the kettles could have formed over buried ice from the melting of ice-moraine cores or preserving subglacial valleys of previous glacial phases as relict long-lived permafrost, which survived even up to 5,000 years after its retreat into the Preboreal. Buried ice from the last glacial period has been found in northern Canada and NW Russia^95^. The relict permafrost was found in NE Poland during drilling in the Suwałki Anorthosite Massif at a depth of less than 350 m b.g.l.^96,97^.

Within the sandur areas, attention was also paid to the so-called pitted sandur (knob-and-kettle sandur), which is evidence of areal deglaciation after the breakup of the glacier front into patches of dead ice, covered with glaciofluvial sediments during the recession^98,99^. These processes are also characteristic of the quiescent phase following the active phase of glacier surge. Due to a strong tension and fracturing of the glacier front, it flattens and separates from the active glacier. Rapid melting covers the glacier front with a dark mineral layer that can insulate such dead ice patches for a long time, favoured by the aggradation of glaciofluvial material. A layer of sediments with a thickness exceeding the depth of the seasonal thawing of the permafrost active layer is sufficient to isolate the dead ice^100^.

Kozarski (1975) indicated a different origin of kettle holes within the Moryń sandur (SW part of Pomerania), who believed they could be of naled ice (aufeis, icings) origin^98^. He motivated this by the occurrence of periglacial conditions in the SIS forefield. Naled ice fields are a typical, indicative phenomenon of the periglacial zones of the Arctic^101^. However, the morphometric analysis of these depressions showed that they are of glacial origin; the maximum depth/diameter ratio was the same as in the case of the subglacial tunnels’ maximum depth/width ratio here^102^. Due to how they are formed, the naled ice fields occupy previously existing depressions in the terrain, so their role, considering the ephemeral nature of the phenomenon, is mainly to preserve the earlier concave landform.

Icelandic studies provide a unique opportunity to track changes in the morphometry of glacial flood-origin kettle holes *in statu nascendi*. The kettle holes are an effective trap for aeolian sediments, constituting an archive of aeolian accumulation rate during the deglaciation of the area up to the vegetation encroachments. The landform’s eolian sediment identification, dating, and depression morphometric changes may be a clue to the kettle hole genesis, also for the Pleistocene kettles environments.

The study aimed to summarise the first three years (seasons 2021/2022–2023/2024) of monitoring the rate of aeolian accumulation within glacial flood-origin kettle holes on the central part of Skeiðarársandur outwash plain in the context of assessing the role of wind, differences in vegetation cover and the morphometry of the depressions as the main factors determining the effectiveness of aeolian processes. The author points to the role of kettle holes as significant sediment traps. Formed as a result of glacial floods, characteristic of marginal zones of glaciers and ice sheets, especially during deglaciation, they constitute a potential archive of sedimentological records between the release of the area from under the ice and vegetation encroachment. It is then, among other things, that an important change in the dynamics of transport and sources of aeolian material occurs. The concept can be used to reconstruct the time window and analogous processes at the end of the Pleistocene in the European Lowlands.

## Study area

### Location

**Fig. 1:**
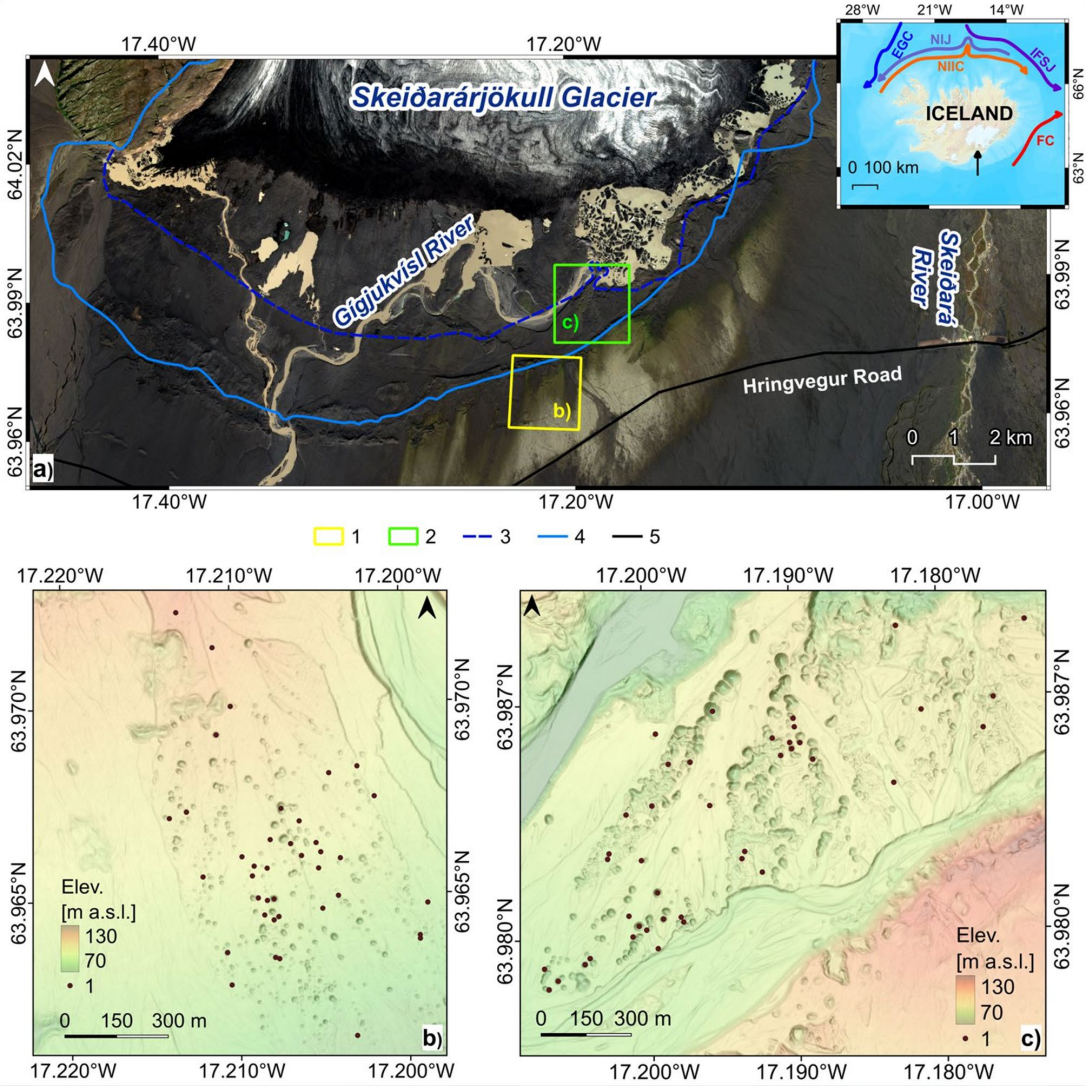
The Skeiðarársandur outwash plain – a study area: (**a**) the marginal zone of the Skeiðarárjökull glacier: 1—the monitored area on an older lever of Skeiðarársandur called Harðaskriða, 2—the monitored area on a younger level after the 1996 jökulhlaup, 3—the Skeiðarárjökull tongue position in 1997, 4—the Skeiðarárjökull tongue position in 1905, 5—the main road, the background—Copernicus Sentinel2 data T27WXM_20240912T125301_TCI (B04-B03-B02 composition bands). Retrieved from Copernicus Browser (https://browser.dataspace.copernicus.eu/) on 7 January 2025, processed by the European Space Agency (ESA), an insert map—main ocean currents around Iceland: EGC – East Greenland Current, NIJ – North Iceland Jet, IFSJ – Iceland-Faroe Slope Jet, NIIC – North Icelandic Irminger Current, FC – Faroe Current (based on^119^), a black arrow indicates the study area; (**b**) the monitored kettle holes within the sandur older level; (**c**) the monitored kettle holes within sandur younger level: 1—kettle holes centroids, the background: ÍslandsDEM, v.1.0 – hypsometric tints with shaded relief (National Land Survey of Iceland; https://atlas.lmi.is/mapview/?application=DEM). Image generated with QGIS 3.34.3 Prizren^120^.

The study area is the central part of the largest active European outwash plain in the Skeiðarárjökull forefield (S Iceland), an outlet valley glacier of the Vatnajökull ice cap (Fig. 1a). It is a zone of massive aggradation of glaciofluvial sediments associated with numerous glacial outburst floods.

Skeiðarárjökull reached its last maximum extent during the LIA, in 1784, 1857, 1873 and 1890–1895^103–105^, aligning its front with the extent of the pushed terminal moraines of that period. It slightly exceeded this extent in several places during the glacier surge in 1929^106^. As a result of the significant glacier recession, a large terminal depression emerged, drained by the Gígjukvísl river. The glacier front is already 2–5.8 km from the LIA moraine line and is still retreating.

Skeiðarársandur outwash plain represents different stages of development regarding relief and the plant colonisation and succession rate. The object of the study was the kettle holes. Some are formed after the melting of buried ice detached from the glacier front^66,107–112^ through englacial hydrofracturing, meltwater conduit collapse and ice cliff collapse^113^. Ice blocks detached in this way, usually the largest fragments, can be buried *in situ*. As they melt, they create extensive depressions up to 20 m deep, marking the glacier’s position. The stepped/concentric ring fractures are a characteristic feature (e.g.^68,113–116^). Until now, these ice blocks have been present under a thick layer of sediments, as confirmed by the GPR research^59^. The average land subsidence rate for large blocks was calculated to be 19.4 ± 2.6 cm yr^–1^ from 1945 to 2017^113^.

However, in most cases, smaller ice blocks are floated to the distal part of the sandur^91,114,117,118^. In shallower water and associated with the vanishing phase of GLOFs, they form characteristic ice-block obstacle marks with sediment shadow. Small blocks, carried by floodwaters further from the glacier edge, melt much faster, even within one ablation season.

The analysed kettle holes on Skeiðarársandur are located on two levels: (1) on the older Harðaskriða (Fig. 1b), formed after various glacial flood episodes from the end of the 19th century to the 1930s^66,113^, with stabilised slopes, often completely covered with mosses and even encroaching willow and birch; (2) on the younger level (Fig. 1c), within the Skeiðarárjökull terminal depression, which experienced a catastrophic jökulhlaup in November 1996^110^, not yet covered with vegetation or with an initial phase of succession, where processes related to the melting of buried ice, mass movements and surface runnof are very dynamic.

### Climate and vegetation

**Fig. 2:**
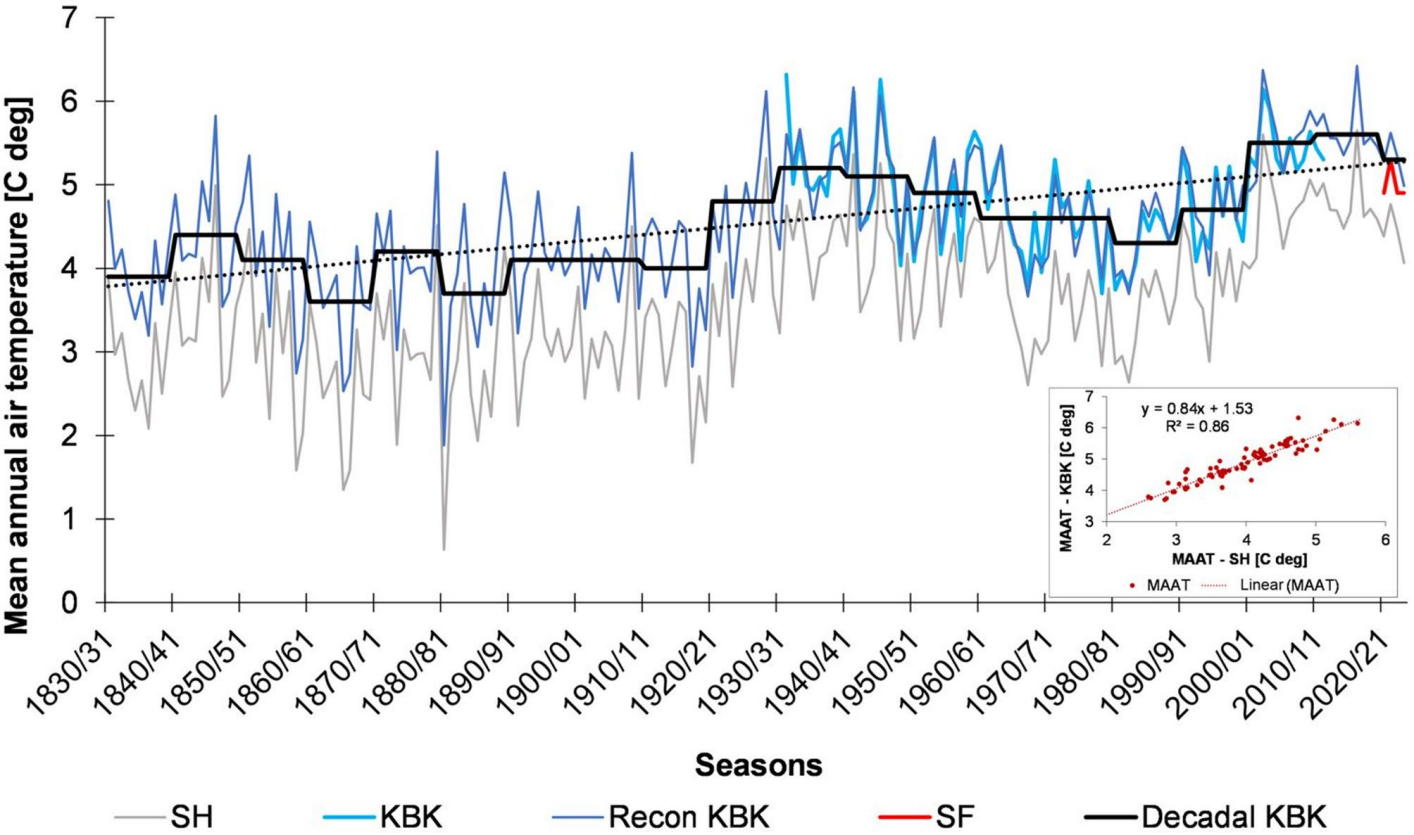
Mean annual air temperatures for Stykkishólmur (SH), Kirkjubæjarklaustur (KBK) (measured, reconstructed and decadal) and Skaftafell (SF) weather stations, 1830/31–2023/24 (based on data of the Icelandic Meteorological Office; https://en.vedur.is/climatology/data/); an insert graph—the relationship between SH and KBK MAATs (*n* = 79, *p* = 0.001). Graphs generated with Microsoft Office LTSC Professional Plus 2021 – Excel^124^.

Nowadays, the cold-temperate oceanic climate of the island is strongly influenced by its location between the warm and the cold ocean currents^119,121,122^ (see Fig. 1a – an insert map) and the position of the polar front^7^. As shown by long-term MAAT data measured and reconstructed for the Kirkjubæjarklaustur (KBK) station (Fig. 2) during the period 1830/31–2023/24, its values ​​ranged from 1.9 °C in the 1880/81 season (the end of the LIA) to 6.4 °C in the 2016/17 season. The average MAAT value for this part of Iceland during the 193 years increased from 3.8 °C to 5.3 °C. Colder periods were the 1860s and 1880s to the 1920s and the 1970–1990s. Warmer periods were especially the 1840s, the 1920–1950s, and the 21st century. Considering the reference period 1991–2020, the average MAAT was 5.3 °C ± 0.5 °C. MAAT values ​​of 5 °C (^123^—**Zenodo_Table1_meteo.pdf**), measured at the Skaftafell (SF) station near the study area for the 2020/21–2023/24 seasons, are close to the average, resulting from the trend. During the year, July is usually the warmest, with an average exceeding 10 °C, although there are also seasons with warmer August or June. The coldest periods are December and January, when the monthly averages from the multi-year period fall slightly below zero.

Stations in southern Iceland (KBK and SF) record significantly lower values ​​of the mean annual wind speed than western Iceland (SH) (^123^—**Zenodo_Meteo_data_KBK.xls** and ^123^—**Zenodo_Fig1_Meteo_graphs.png**). The multi-year values ​​for KBK are 3.6 m s^–1^ ± 0.6 m s^–1^. The period from the 1980s to the beginning of the 21st century was particularly windy, with a maximum of 5.4 m s^–1^ in the 1988/1989 season. Recent years, however, have been marked by lower values ​​– at the SF station in the 20s of the 21st century, the average was 2.7–3.0 m s^–1^. During the year, it is windiest from December to April, with a maximum in January and the calmest in July and August.

The south coast receives high rainfall values, which in the multi-year period 1931/32–2011/12 in KBK were 1,722 mm ± 260 mm, and in 1964/65–2021/22 in SF were 1,582 mm ± 280 mm. The 21st century has been wetter regarding rainfall totals, similar to the 1940s and 1950s. The rainiest month is October, when monthly rainfall totals exceed 160 mm, and the most “dry” is May, when the average monthly rainfall total in KBK was 113 mm and in SF, 86 mm.

Climate features are strongly related to vegetation. The marginal zone of the Skeiðarárjökull glacier is currently undergoing intensive deglaciation, which is why pioneer vegetation, tundra dominated by moss and dwarf heaths, is the first to enter^7,125^. However, almost the entire southern coast is a zone of subarctic birch forest^7^. It is reflected in the rapid entry of *Betula pubescens* and hybridisation between this species and *Betula nana*^126^, characteristic of periods of pronounced warming. The growing season on Skeiðarársandur, calculated as the longest continuous period with an average daily air temperature above 5° C, ranged from 98 to 136 days in 2021/2022–2023/2024, from approximately the end of April/mid-May to the second half of September.

## Data and methods

All data and figure files supplementing this publication are available in the open international data repository Zenodo^123^ and are cited below in the text (“Zenodo” in the file name). The rest of the data can be found as **Supplementary Material**.

### Meteorological data

These data come from the Icelandic Meteorological Office (IMO; https://en.vedur.is/climatology/data/) and concern three stations: Stykkishólmur (SH), located in W Iceland, 13.18 m a.s.l. (WMO number 4,013), Kirkjubæjarklaustur (KBK) in S Iceland, 22 m a.s.l. (WMO number 4,064) and Skaftafell (SF), located about 75 km NEE of KBK, 86 m a.s.l. (WMO number 4,172). The SH station’s annual and monthly mean air temperature values have the longest measurement series. They are available for the period 1832–1999 (https://en.vedur.is/Medaltalstoflur-txt/Stykkisholmur.txt), and the data with other parameters cover the period 1949–2024 (https://www.vedur.is/Medaltalstoflur-txt/Stod_178_Stykkisholmur.ManMedal.txt). They were used to reconstruct temperature changes at KBK, the station closest to the study area with the longest meteorological data series. The reconstruction was based on the linear regression equation with R^2^ = 0.86 (*n* = 79, *p* = 0.001) (cf. Figure 2 – an insert graph and ^123^—**Zenodo_Meteo_data_KBK.xls**). Data for this station are from 1931 to 2013 (https://vedur.is/Medaltalstoflur-txt/Stod_772_Kirkjubajarklaustur.ManMedal.txt).

Thanks to the courtesy of IMO, hourly data and daily values ​​for the SF station from June 2020 to June 2024 were also obtained (^123^—**Zenodo_Meteo_Skaftafell_2020–2024.xls** and ^123^—**Zenodo_Fig2_Meteo_Skaftafell_graphs.png**). This station is located about 16 km NE of the study area. For monthly precipitation totals in SF, data are available for the period 1964–2023 (https://vedur.is/Medaltalstoflur-txt/Stod_748_Skaftafell.ManMedal.txt; see also ^123^—**Zenodo_Fig1_Meteo_graphs.png**). They were statistically analysed and visualised as graphs in Excel (Microsoft Office LTSC Professional Plus 2021).

### Sentinel satellite scenes

The tundra vegetation of Iceland is most developed in mid-summer, from mid-July to late August^127^. Therefore, I tried to find Sentinel-2-L2A satellite scenes with the acquisition date close to this time. The Copernicus Browser (ESA) (https://browser.dataspace.copernicus.eu/) was used for this purpose. The following data were downloaded on 7 January 2025: S2A_MSIL2A_20210717T124311_N0301_R095_T27WXM_20210717T152018 from July 17, 2021, S2A_MSIL2A_20220804T125311_N0400_R138_T28VCR_20220804T191757 from August 4, 2022, and S2A_MSIL2A_20230819T125311_N0509_R138_T27WXM_20230819T153951 from August 19, 2023. The data were initially prepared in the WGS 84/UTM zone 27 N coordinate reference system (CRS) (EPSG:32627) and converted to the ISN93/Lambert 1993 system (EPSG:3057).

### Digital elevation model

DEM with a resolution of 2 m × 2 m were downloaded from the National Land Survey of Iceland (Landmælingar Íslands) – ÍslandsDEM, v.1.0: https://atlas.lmi.is/mapview/?application=DEM. These were two files in the ISN93/Lambert 1993 CRS (EPSG:3057) with grid numbers 61 and 62. They came from the airborne lidar technology campaign in 2012^128^. The data are publicly available under the Attribution 4.0 International (CC BY 4.0) license.

### Preliminary work

**Fig. 3:**
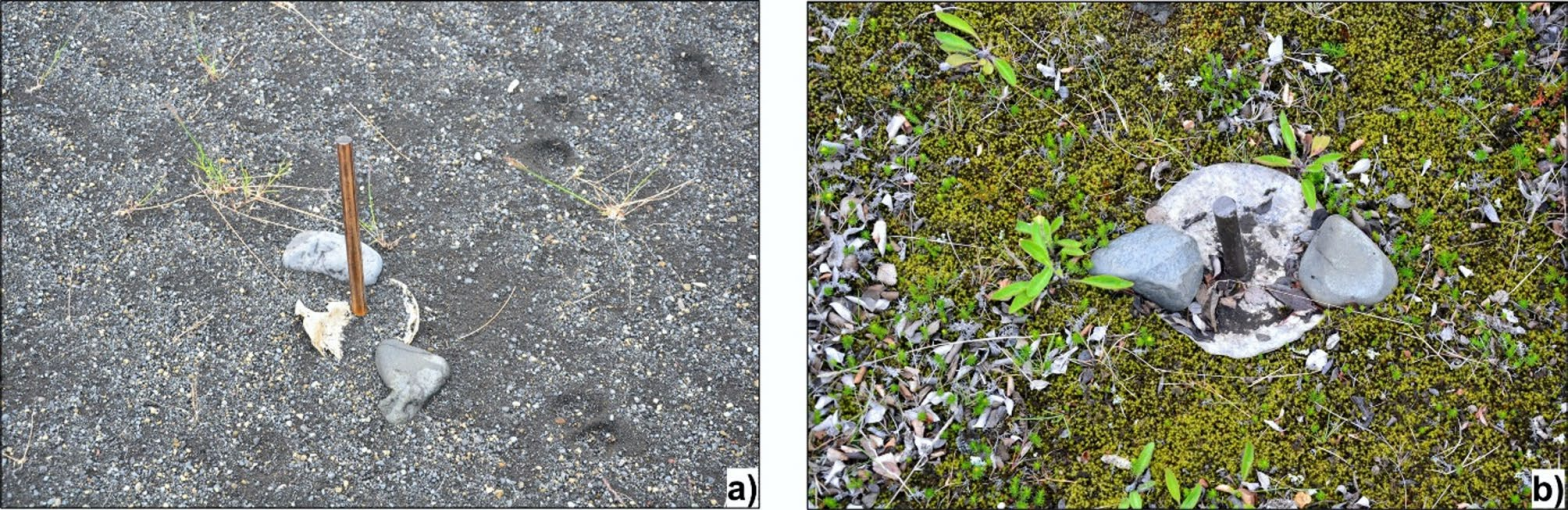
Examples of aeolian accumulation monitoring sites on Skeiðarársandur – wooden stakes with cardboard plates and pebbles: (**a**) the kettle hole no. ZY19 on the younger level – a plate with sediments, 23.06.2023 (installed on 24.06.2022); (**b**) the kettle hole no. ZOW06 on an older level – a plate with mineral and organic sediments, 20.06.2024 (installed on 26.06.2023). Phot.: Joanna E. Szafraniec.

**Fig. 4:**
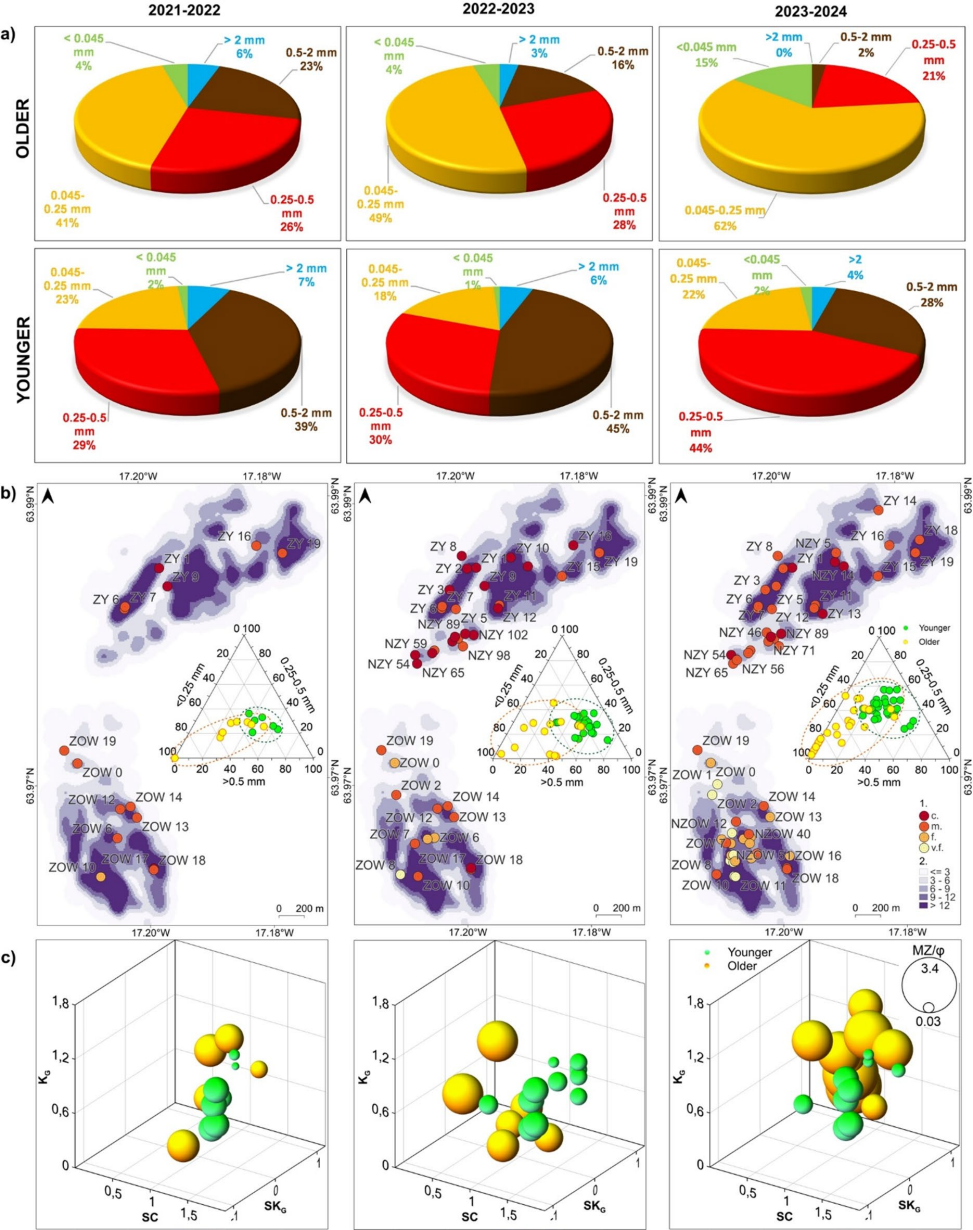
Statistical and spatial characteristics of mineral aeolian sediments within kettle holes on Skeiðarársandur in 2021/22–2023/24: (**a**) a granulometric composition; graphs generated with Microsoft Office LTSC Professional Plus 2021 – Excel^124^; (**b**) mean size classes of aeolian mineral grains against the background of the density of the examined kettle holes and the share of the three main fractions [%] in the granulometric composition for each depression (ternary scatter plots): 1—particle mean size class where c. – coarse sand, m. – medium sand, f. – fine sand, v.f. – very fine sand, 2—density of kettle holes [number of depressions per 100-meter radius area]; maps generated with QGIS 3.34.3 Prizren^120^ and ternary plots with Golden Software LLC – Grapher 11^140^; (**c**) mean grain size [MZ in φ units] against the background of relationship between sorting coefficient (SC), skewness (SK_G_) and kurtosis (K_G_) of data; 3D bubble plots generated with Golden Software LLC – Grapher 11^140^.

Before starting field observations, the kettle holes were first catalogued – 1,036 landforms within the younger outwash level and 757 landforms within the older level (^123^—**Zenodo_Younger-KH_codes.zip**, ^123^—**Zenodo_Older-KH_codes.zip**, ^123^—**Zenodo_Fig3_Younger-KH.png** and ^123^—**Zenodo_Fig4_Older-KH.png**). ÍslandsDEM, v.1.0 was used for this purpose. Three methods of visualising the relief were combined: hypsometric tints, dense contour lines with a cut of 0.1 m (closed isolines) and combined hill-shading at a light azimuth of 315° and light altitude of 45°. On this basis, in the QGIS software^120^ (different versions), the shape of the depressions was manually vectorised, defining their boundaries. The program also calculated basic morphometric parameters of depressions, such as their planar area, perimeter, maximum depth, volume, average depth and heights: maximum, minimum and average (^123^—**Zenodo_Morph_KH_Younger.xls** and ^123^—**Zenodo_Morph_KH_Older.xls**). The Lambert Conformal Conic projection handles shapes and angles well. Since the landforms studied were small (average diameter of 12.7–18.8 m) and shallow (average maximum depth of 1.3–2.5 m), with a DEM resolution of 2 × 2 m, any distortions resulting from the projection are negligible for morphometric analysis. Based on the kettle holes centroids, their density was also calculated within a radius of 100 m by the Heatmap option (Kernel density estimation) in QGIS. The next step was randomly selecting 20 depressions within both outwash levels to initiate monitoring of the aeolian accumulation rate. Vector research tools in QGIS (Random Points Inside Polygons) were used.

### Fieldwork research

Wooden stakes 0.5 m long were installed in the selected kettle holes on both levels of Skeiðarársandur in June 2021. The stakes were driven into the bottom, in its lowest part, and outside the reach of landslides and slope runoff. A cardboard plate 0.15 m in diameter with a hole of 0.015 m in diameter was placed on the stakes. To prevent the plate from being carried away by the wind, it was attached with larger boulders so that the stones occupied c. 10% of the plate’s surface (Fig. 3). The stake height was also measured with an accuracy of ± 0.001 m. The stakes on the younger level were given the ZY signature, and on the older one, the ZOW.

The advantage of this method is its simplicity and low cost. The limitation is the plate durability for one, a maximum of two seasons. In addition, the cardboard in places not held by pebbles tends to bend during drying, which is the most significant drawback of the method. A proposal to bring the conditions closer to natural ones may be to install a rough mesh sanding disc with the diameter of the plate and a wooden dowel passing through the stake, holding the plate, which also allows the exclusion of pebbles (^123^—**Zenodo_Fig10_Roughness.png**). This idea was initially verified in the 2023/2024 season for the NZY65 kettle on the younger level and ZOW08 on the older one. The sediment samples in the NZY65 kettle were similar in total weight; on the mesh sanding disc, the mass was lower than the mass on the plate without the mesh (119.6 g and 123.2 g, respectively). In the ZOW08 kettle, the sediment sample on the mesh was heavier than on the plate without the mesh (2.7 g and 2.5 g, respectively). A higher share of coarser fractions was noted on the plate with pebbles (grain diameter ≥0.25 mm) and, in turn, a higher share of finer fractions on the plate with the mesh (grain diameter < 0.25 mm). The mesh also prevented the plate from bending. Errors related to the method’s effectiveness based on the experiment using a rough mesh disc are estimated at ± 3–8%. However, this issue requires further research.

It was assumed that the aeolian accumulation would be monitored for 12 months until June of the following year. After a year, the plate and its contents were removed and packed into a bag with the signature description, sample number and date. In June 2022 and 2023, plates with a diameter of 0.18 m were placed, and the surface occupied by pebbles was increased to 20% (see Fig. 3) because, in the first observation season, some of the plates were carried away by the wind. In addition, the number of stakes was increased to 40 pieces on each level. The increase in stakes was dictated by the fact that during the winter seasons, snow accumulates in the kettle hole, causing some of the boulders to move. After the snow melts, the exposed and unsecured plates can be carried away by the wind. New stakes on Harðaskriða were marked with the signature NZOW and on the younger level NZY. In the case of new stakes installed in 2022 at the older outwash level (except NZOW23 and 28), due to the negligible amount of material, sediment collection in 2023 was abandoned, leaving them until 2024. The location of all observation sites is presented in Fig. 1b, c. Data on the observation results were posted in the Zenodo repository, taking into account the annual mass of sediments, annual accumulation rate, annual thickening rate, granulometric composition (^123^—**Zenodo_aeol-acc_OlderKH.zip** and ^123^—**Zenodo_aeol-acc_YoungerKH.zip**). Regarding average area ​​and maximum depth values, the morphometric parameters of the kettle holes selected for the observation network fall between the median and the arithmetic mean, so they can be considered representative of the entire population of catalogued depressions.

## Data analysis methods

### Mineral grain characteristics of aeolian material

All sediments were dried and separated from the cardboard plates in the Soil and Rock Analysis Laboratory of the University of Silesia in Katowice. They were then weighed on an Axis electronic analytical scale with a reading accuracy of 0.1 g. The predominant sediment was sand, consisting mainly of volcanic rocks with small admixtures of other rocks and plant macroparticles. After weighing, all samples were sieved. It resulted in the division into fractions: gravel (> 2 mm of diameter), coarse-grained sand (2–0.5 mm), medium-grained sand (0.5–0.25 mm), fine-grained sand (0.25–0.045 mm) and silt-clay fraction (< 0.045 mm of diameter). The sediment of each fraction was weighed separately (see ^123^—**Zenodo_aeol-acc_OlderKH.zip** and ^123^—**Zenodo_aeol-acc_YoungerKH.zip**). At the sieving stage, organic macroparticles were also separated, i.e. grass blades, birch and willow leaves and their dry inflorescences, heather twigs, crowberry berries and various seeds.

In the next step, basic statistical measures for mineral particles were calculated. These were the mean particle size (MZ in φ units), sorting coefficient (SC), skewness (SK_G_) and kurtosis (K_G_), calculated from formulas by^129,130^ (formulas 1–4). The main components of the equations were based on the cumulative percentage share of a given fraction. They concerned the average values ​​of the particle diameter, constituting 5% (φ5), 16% (φ16), 25% (φ25), 50% (φ50), 75% (φ75), 84% (φ84) and 95% (φ95) of this share (**Supplementary Table ****S1**). The φ value was calculated from the formula: $\:\phi\:=\:-{log}_{2}\:D$, where D is the particle diameter in mm^129^.

1 $$\:\text{M}\text{Z}\:=\frac{\phi\:16+\phi\:50+\phi\:84}{3}$$

2 $$\:\text{S}\text{C}\:=\frac{\phi\:84-\phi\:16}{4}+\frac{\phi\:95-\phi\:5}{6.6}$$

3 $$\:\text{S}\text{K}\text{G}\:=\frac{\phi\:16+\phi\:84-2\phi\:50}{2(\phi\:84-\phi\:16)}+\frac{\phi\:5+\phi\:95-2\phi\:50}{2(\phi\:95-\phi\:5)}$$

4 $$\:\:\text{K}\text{G}\:=\frac{\phi\:95-\phi\:5}{2.44(\phi\:75-\phi\:25)}$$.

The division of samples into sorting classes of the material was performed using the^130^ classification based on SC. The following classes were distinguished: SC < 0.35 – very well sorted, 0.35–0.50 – well sorted, 0.50–0.71 – moderately well sorted, 0.71–1.00 – moderately sorted, 1.00–2.00 – poorly sorted, 2.00–4.00 – very poorly sorted and SC > 4.00 – extremely poorly sorted. In addition, graphs presenting the grain size cumulation curves within each kettle hole of both sandur levels were prepared. Inflexion points FT on the curves, related to the change in the line inclination angle (45°), were the basis for determining the percentage share of grains in a given mode of their transport^131,132^ (**Supplementary Table** **S2**).

### Aeolian accumulation and thickening rate

The annual thickening layer of aeolian sediments in mm yr^–1^ was calculated by dividing the accumulation rate [g cm^–2^ yr^–1^] by their bulk density [g cm^–3^ yr^–1^]. Different density values ​​were used for both levels due to differences in the organic content. For the younger outwash horizon, 17 sediment samples (^123^—**Zenodo_Accumulation-vs-Density.xls**) were selected, the mass of which was higher than 200 g (including organic matter) and for which the thickness of the annual sediment layer measured in the field was also known (the plate was covered entirely with sediments). The sediment density in g cm^–3^ for each sample was calculated from the ratio of the sediment mass to the product of its layer’s annual thickness and the plate’s net area. The average sediment’s bulk density was 1.1 g cm^–3^ (median) with an interquartile range (IQR) of 0.8–2.3 g cm^–3^. Such a variation in values ​​was probably due to different proportions between porous lava fragments and other types of rocks. In the case of the older horizon, where such calculations were impossible due to the negligible sediment mass, a different approach was used. For areas in the vegetation cover class 2–6 (description of vegetation classes in the further part of the **Methods** section), the bulk density was used with the same value as for the younger horizon, i.e. 1.1 g cm^–3^. In the area with vegetation classes above 6, the data from^43^ for organic content above 10%, i.e. 0.6 g cm^–3^, were used. In the **Results** section, accumulation rate values ​​were given in g m^–2^ yr^–1^.

### Vegetation coverage

Sentinel 2 satellite scenes were used to calculate the area covered by vegetation and the Green Chlorophyll Index (GCI) and Normalized Difference Vegetation Index (NDVI). The GCI index assesses the plant’s chlorophyll content. It is susceptible to light and nutrient availability, water stress and vegetation health^133^, thus also indirectly reflecting the tundra conditions in a given season. The GCI was calculated in the QGIS raster calculator tool using the formula: NIR/GREEN – 1 (https://www.indexdatabase.de/db/i-single.php?id=128), where NIR is the Near Infrared band; here B08 (833 nm of central wavelength) and GREEN is the green band; here B03 (559 nm). The NDVI index assesses the biomass content, vegetation health and photosynthetic efficiency. It also shows a dependence on environmental factors related to land use, its altitude, or slope inclination and exposure^134^. The B08 and RED bands were used for calculations; here, B04 (665 nm of central wavelength) and the formula: (NIR – RED)/(NIR + RED). All the above-mentioned bands have a resolution of 10 m.

**Fig. 5:**
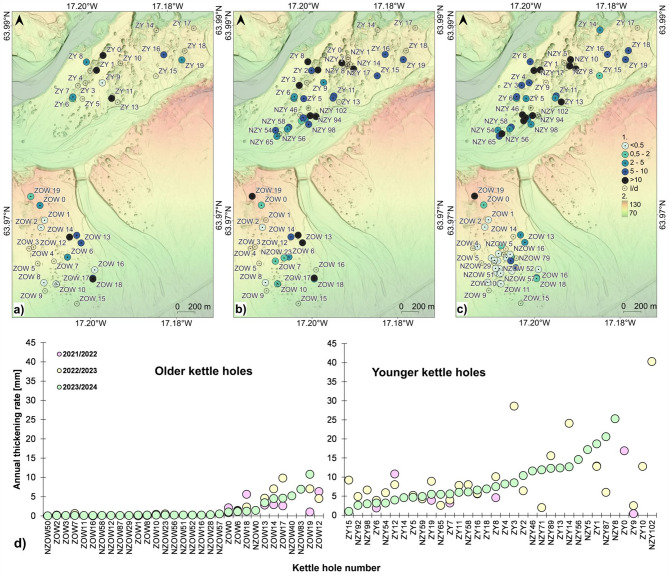
Spatial distribution of annual thickening rate of aeolian sediments within kettle holes on both levels of Skeiðarársandur: (**a**) in June 2021–June 2022; (**b**) in June 2022–June 2023; (**c**) in June 2023–June 2024: 1—value ranges in mm yr^–1^, 2—an elevation in metres above sea level, the background— ÍslandsDEM, v.1.0 – hypsometric tints with shaded relief (National Land Survey of Iceland; https://atlas.lmi.is/mapview/?application=DEM); (**d**) a value ranking of the annual thickening rate of aeolian sediments, in 2021/22–2023/24, on older and younger levels of Skeiðarársandur sorted by values of the third season. Maps generated with QGIS 3.34.3 Prizren^120^. Graphs generated with Microsoft Office LTSC Professional Plus 2021 – Excel^124^.

For each season, rank abundance curves (^123^—**Zenodo_GCI_NDVI_Vege-classes.xls**) were developed for both indexes. They were used to assign ranks regarding vegetation intensity in the study area, including biomass (NDVI) and chlorophyll contents (GCI). The division into ranks was made based on natural inflexion points on the curves. Individual divisions were assigned ranks from 1 (no vegetation) to 6 (the highest biomass/chlorophyll content). Finally, the ranks of both indicators were summed, obtaining classes: 2–4 – lack or sparse vegetation (also water and ice bodies), 5–6 – low level of vegetable coverage, mainly tufts of grass and moss, 7–8 – medium level of vegetation coverage, 9–10 – high level of vegetation coverage and 11–12 – entirely covered by dense vegetation (^123^—**Zenodo_Fig5_Method_Vege-classes.png** and ^123^—**Zenodo_Table2_Vege-classification.pdf**).

**Fig. 6:**
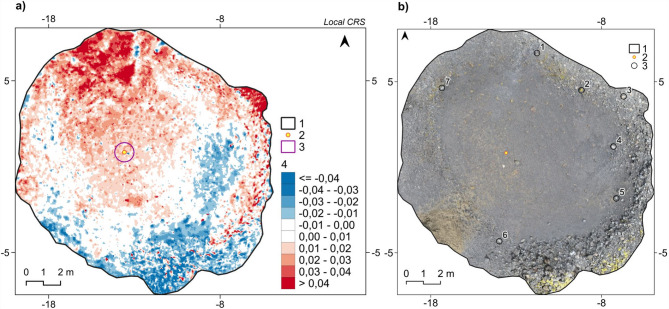
Results of the photogrammetric survey for the ZY05 kettle hole (a younger level of Skeiðarársandur;^123^—**Zenodo_ZY05_data_SfM.zip**): (**a**) DEM of Difference between June 2022 and June 2024 with the resolution of 0.05 m × 0.05 m; 1—a kettle border, 2—a stake with a plate location, 3—an area of the thickening value calculations, 4—the differences in metres; (**b**) an area coverage of the kettle hole in June 2024; 1—a kettle border, 2—a stake with a plate location, 3—the Ground Control Points location. Image generated with QGIS 3.34.3 Prizren^120^.

To determine the percentage of vegetation cover in uniform test areas, colour infrared composites were created based on 10 m resolution bands: B08, B04 and B03. The Built virtual layer option in QGIS (^123^—**Zenodo_Fig6_Method_Vege-cover.png**) was used for this purpose. In the image created this way, the area covered with vegetation is distinguished from other land cover forms by different red shades. In order to automatically extract “vegetation” rasters from satellite scenes, the unsupervised classification tool K-Means Clustering for Grids was used in SAGA GIS 7.8.2^135^, and the creation of 25 clusters was assigned. The classification grid was then converted to polygons. The vegetation cover polygons (i.e. those corresponding with red rasters) were merged using the dilation and erosion operation in QGIS^136^, using positive (+10 m) and negative (–10 m) buffers. For each depression, the area occupied by vegetation within a radius of 100 m from its centroid was calculated. The vegetation cover coincides with the range of the last three classes generated using the GCI and NDVI indices. The GCI, NDVI and vegetation cover values are included in^123^—**Zenodo_aeolian-acc_OlderKH.zip** and ^123^—**Zenodo_aeolian-acc_YoungerKH.zip** packages.

### Wind effect index

**Fig. 7:**
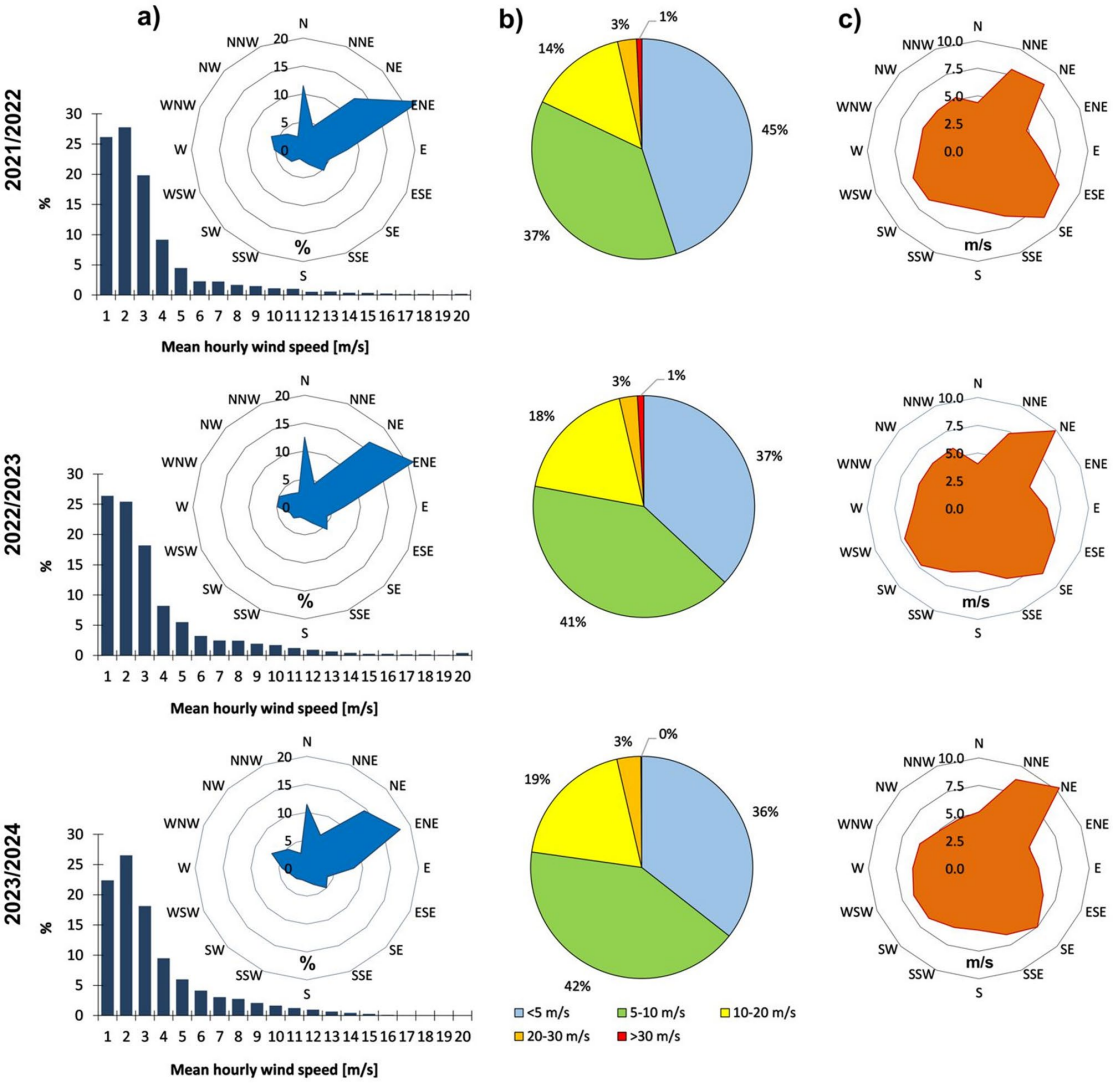
Characteristics of wind conditions on both levels of Skeiðarársandur in 2021/22–2023/24: (**a**) distribution of cases with mean hourly wind speed [m s^–1^] (histograms) and mean hourly wind speed distributed according to cardinal wind directions (wind rose graphs); (**b**) percentage share in classes of maximum hourly wind gusts; (**c**) average speed of maximum hourly wind gusts distributed according to cardinal wind directions (based on data from Icelandic Meteorological Office—https://en.vedur.is/climatology/data/;^123^;—**Zenodo_Meteo_Skaftafell_2020–2024.xls**). Graphs generated with Microsoft Office LTSC Professional Plus 2021 – Excel^124^.

The 2 m resolution DEM was also used to calculate the Wind Effect Index (Windward/Leeward Index), a tool in the SAGA GIS program (Terrain Analysis tools = > Morphometry). Values ​​of this index above 1 indicate a significant role of wind in shaping the surface (wind exposure), while values ​​below 1 indicate wind shadow areas. The advantage of the index is that it allows for the assumption during calculations in which direction the wind is blowing (^123^—**Zenodo_Fig7_Wind-effect-index.png**).

### Data from the photogrammetric survey

Data from the Structure from Motion (SfM) technique were used to compare the thickening rate ​​obtained for the ZY05 kettle hole from June 2022 to June 2024. The methodology for obtaining data in the field and the software was described in detail by^137^, and the 2022 data were published in the Zenodo repository^138^. The data used in this study are included in the^123^—**Zenodo_ZY05_data_SfM.zip** package. To generate DTMs with a resolution of 0.05 m × 0.05 m using Multilevel B-spline Interpolation in SAGA GIS, 572,324 points from 2022 (average 8.2 points per model raster) and 682,649 points from 2024 (average 9.8 points per model raster) were used. The data were processed in the local Cartesian coordinate system in metres, defined as the user CRS: +proj = cart + a = 1 + b = 1 + units = m + no_defs. The DTMs were oriented north in the MeshLab program^139^ (Filters = > Normals, Curvatures and Orientation = > Transform: Rotate) based on the azimuths between stakes obtained from the closed traverse method made during the fieldwork research in 2024. The reference level for the height is the value of 0 m of the depression bottom in 2024, i.e. the lowest model point.

## Results

### Aeolian accumulation in kettle holes in the 2021/2022–2023/2024 seasons

During the analysed period, 0 to 11,887 g m^–2^ yr^–1^ sediment accumulated on the depressions bottom of the older outwash level (Harðaskriða). An average value was between 168 and 832 g m^–2^ yr^–1^, and IQR ranges from 70 to 5,006 g m^–2^ yr^–1^ (^123^—**Zenodo_Table3_Acc_rate.pdf**). On the younger horizon, from 451 to 44,282 g m^–2^ yr^–1^ of aeolian material accumulated, with an average value of 5,024–7,415 g m^–2^ yr^–1^, and 50% of samples in the range of 3,569–13,427 g m^–2^ yr^–1^ (IQR). Comparing both levels by the arithmetic mean, the older depressions accumulated 4.3–7.2 times less, on average, wind-borne material by weight than the younger kettles and taking into account the median, 6.0–9.6 times less in the first two seasons and 44 times less in the third season.

It was calculated that in the sediments of the younger horizon, almost the entire content, on average 99.6%, with 50% of values ​​falling within the range of 99.3–99.8%, is mineral particles. The organics are usually single blades of grass or dry fruits and seeds. Within the older horizon, macroscopic organic parts constitute an average of 25.4% of the mass of aeolian sediments, of which 50% ​​(IQR) fall within the 11–57% range. These are mainly leaves and twigs of birch and willow, fragments of heather, crowberries, seeds, and fruits.

If only the mineral parts were considered, sand dominates. In the Harðaskriða depressions, the sand fraction constitutes 86–98% of the mineral composition (Fig. 4a, b), of which the most is fine-grained sand (41–62%) and medium-grained sand (21–28%) (see ^123^—**Zenodo_aeolian-acc_OlderKH.zip**). The content of the gravel fraction is variable and ranges from 0 to 6%, and the silt/clay fractions are about 4–15%. Coarser-grained sand accumulated in the eastern part of the area (Fig. 4b), and finer sand in the central part, along the oriented former bar of landforms with a higher density per unit area (see also **Supplementary Fig. ****S1**). The median value of MZ in φ units is 1.28 (from 0.70 to 2.74) in the first season (average diameter of 0.42–0.50 mm – medium sand), 1.49 (from 0.70 to 3.32) in second (average diameter of 0.35–0.42 mm – medium sand) and 2.30 (from 0.26 to 3.32) in the third season (average diameter of 0.177–0.21 mm – fine sand) (Fig. 4c, **Supplementary Table ****S1**). Grains of the older horizon are poorly sorted, and also moderately sorted in the third season; the median value of SC is 1.33 in the first season (0.26–1.76), 1.23 in the second (0.79–1.76), and 0.90 in the third (0.26–1.93).

Within the younger kettle holes, the sand fraction also dominates in the aeolian sediments, constituting 91–94% of the composition (Fig. 4a, b). These are especially coarse-grained (28–45%) and medium-grained (29–44%) sands (see ^123^—**Zenodo_aeolian-acc_YoungerKH.zip**). The gravel fraction constitutes approximately 4–7% of the composition, and the finest fractions constitute 1–2%. There is no clear spatial pattern in terms of MZ size class (Fig. 4b). The median value of MZ in φ units is 1.28 (from 1.00 to 1.33) in the first season (average diameter of 0.35–0.42 mm – medium sand), 0.70 (from 0.26 to 1.28) in the second (average diameter of 0.30–0.35 mm – medium sand) and 1.28 (from 0.26 to 1.30) in the third season (medium sand). The collected samples are also poorly and moderately sorted (**Supplementary Table ****S1**); average SC is 1.28 (1.00–1.33), 1.33 (0.90–1.33) and 1.23 (0.90–1.50), respectively, in each season.

The graphs presenting the nature of the fraction distribution (Fig. 4c; see also **Supplementary Table ****S1**) for sediment samples from the older sandur level show a shift towards negative skewness in two first seasons, which indicates large proportion of coarse particles^129^, and positive skewness in third season – fine particles, especially in the western part of the area. At the same time, a median value of kurtosis is 0.5–0.6 in the first two seasons, indicating a very platykurtic distribution of data (a low degree of particle size concentration around the average value), and 0.95 in the third season – mesokurtic distribution. In the case of the younger sandur level, a shift in the distribution towards negative skewness is noticeable in the first and third seasons (coarse particles), and towards strongly positive skewness in the second season, indicating a higher share of finer fractions. The median kurtosis value is about 0.69–0.72, indicating the platykurtic distribution of data.

### Thickening rate of aeolian sediments at Skeiðarársandur

At the older Skeiðarársandur level, the accumulation rate ranged from 0 to 10.8 mm yr^–1^ of aeolian sediment during the period 2021/2022–2023/2024, with an average value of 0.2–1.4 mm yr^–1^, of which 50% of the values ​​(IQR) were between 0.1 and 4.6 mm yr^–1^ (^123^—**Zenodo_Table4_Thick_rate.pdf**). The accumulation rate at the younger outwash level ranged from 0.4 to 40 mm yr^–1^. The median was 4.6–6.8 mm yr^–1^, with IQR between 3.2 and 12.2 mm yr^–1^. On average, the older outwash level accumulates a 3.3–6.3 times thinner layer of material per year than the younger level, and in terms of the median, 3.3–5.1 times thinner in the first two seasons and 34 times thinner in the third season.

While individual seasons differ in values ​​(Fig. 5), the spatial pattern of the accumulation rate is repeated from year to year – the values ​​are higher in the younger outwash level. The older level is dominated by landforms with annual aeolian accumulation below 0.2 mm yr^–1^, and higher values ​​occur in the kettle closest to the edge descending to the terminal depression with the younger level and in the eastern part. The younger level is dominated by landforms with accumulation above 4 mm yr^–1^, and lower values ​​occur mainly on the southern edge of the depression zone and the esker top (cf.^123^—**Zenodo_aeolian-acc_OlderKH.zip** and ^123^—**Zenodo_aeolian-acc_YoungerKH.zip**).

The ongoing monitoring of the aeolian accumulation provides point information relating to a small fragment of the kettle bottom. In parallel, spatial monitoring is also conducted using the Structure from Motion (SfM) technique^137^. Reference material was prepared, obtained in June 2022^138^, i.e. point clouds and high-resolution digital terrain models (DTMs) for selected kettle holes. Subsequent years of observation provide further data (in preparation). An example is the ZY05 kettle at a younger level (^123^—**Zenodo_ZY05_data_SfM.zip**). The calculated DEM of Difference (DoD) with a resolution of 0.05 m × 0.05 m between June 2022 and June 2024 allowed me to estimate (Fig. 6a) that on the surface of 1 m^2^ around the stake, the average difference was 15 mm ± 9 mm. The measured thickening rate at that time on the plate was 9.9 mm ± 2 mm. Over the two seasons, −0.76 m^3^ of sediments were blown out of the depression, and +1.91 m^3^ of sediments were deposited. It gives a net volumetric aeolian accumulation of +1.15 m^3^ ± 0.45 m^3^. For the entire analysed depression, this is an average thickening rate of 7 mm over the two seasons. The obtained DoD also shows that the accumulation rate is not spatially uniform but dominated by the prevailing NE wind. Deposition occurred on the southern-facing slopes, on the eastern and western edges and most of the landform’s bottom surface. Wind-blowing dominated on the northern-facing slopes and at the eastern bend of the bottom and slopes, making these parts of the depression rougher (Fig. 6b).

### Wind conditions and aeolian accumulation rate

A wind speed of ~ 3–5 m s^–1^ is sufficient to lift dust and sand particles from the ground^28^. All three seasons were characterised by wind conditions close to the average for the reference period 1990–2020, reaching an average wind speed of 2.7 m s^–1^ in the 2021/2022 season and 3.0 m s^–1^ in the 2022/2023 and 2023/2024 seasons. A characteristic feature was the dominance of hourly wind speeds (based on the 10-min. average) in the range of up to 3 m s^–1^, which accounted for 73.8% of the time in the first season, 70% in the second season and 67% in the 2023/2024 season (Fig. 7a). The duration with wind in the ranges of 4–6 m s^–1^ increased, by 15.9%, 16.9% and 19.6%, respectively, with each season. Hourly series with mean wind speed above 5 m s^–1^ in all seasons lasted on average 4 h; in 2021/2022, there were 139 series (average max. wind gusts 7.5 m s^–1^ ± 2.4 m s^–1^), in 2022/2023 – 177 series (average max. wind gusts 7.4 m s^–1^ ± 1.9 m s^–1^), and in 2023/2024 – 187 series (average max. wind gusts 7.1 m s^–1^ ± 1.9 m s^–1^). It is consistent with the findings of^141^, who estimated that there may be 101 dust days per year in southern Iceland. The longest hourly series with average wind speed above 5 m s^–1^ lasted 45 (5–7.04.2022), 78 (19–22.12.2022) and 136 h (27.03–1.04.2024), respectively. In the first season, there were six such series lasting more than 24 h (average of 32.2 h); in the second – eight (average of 41.5 h); in the third – seven such series (average of 54.3 h). The ENE and NE sectors were the dominant wind direction, constituting 35.5%, 37.5% and 32.6% of the time in the subsequent seasons. The wind blew least frequently from the SSE, S, SSW, and SW sectors – 9.5%, 10% and 10.5% of the time in the subsequent seasons.

In terms of maximum wind gusts within an hour (based on 3 s. values), all seasons were similar for speeds above 20 m s^–1^ and constituted 3–4% of the time (Fig. 7b), although in the third season, no gusts above 30 m s^–1^ were recorded. The 2021/2022 season was the mildest when maximum gusts below 5 m s^–1^ lasted 45% of the time. In the 2022/2023 and 2023/2024 seasons, the dominant time was when maximum gusts above 5 m s^–1^ during the day constituted 41–42% of the year and above 10 m s^–1^ – 18–19%. The strongest hourly wind gusts occurred with the wind from the NE sector, reaching an average of 8.5–10.3 m s^–1^ and from the SE sector – 7.5–8.5 m s^–1^ (Fig. 7c). The maximum hourly gusts from the N sector were the weakest, reaching an average of 4–5.1 m s^–1^.

If we compare the annual aeolian accumulation data in the kettle holes (cf. Figure 5d), in which measurements were made in all three seasons (17 such depressions), in 2021/2022, the total accumulation value was the lowest, constituting 26% of the aeolian material. The second and third seasons were similar regarding wind conditions. Aeolian accumulation constituted 39% and 35%, respectively. The second period’s advantage could have been the higher average wind speed during the hourly series with speeds above 5 m s^–1^, including episodes of maximum wind above 30 m s^–1^ (maximum of 48 m s^–1^). Based on meteorological data (see ^123^—**Zenodo_Meteo_Skaftafell_2020–2024.xls**), it was found that at higher wind speeds, the series with average speeds above 5 m s^–1^, i.e. critical speeds for the wind deflation potential, also lengthen – from 2 hours with an average wind of 6.6 m s^–1^ to 45 h with average speeds of 11.1 m s^–1^ (R^2^ = 0.83, *n* = 15, *p* = 0.001).

**Fig. 8:**
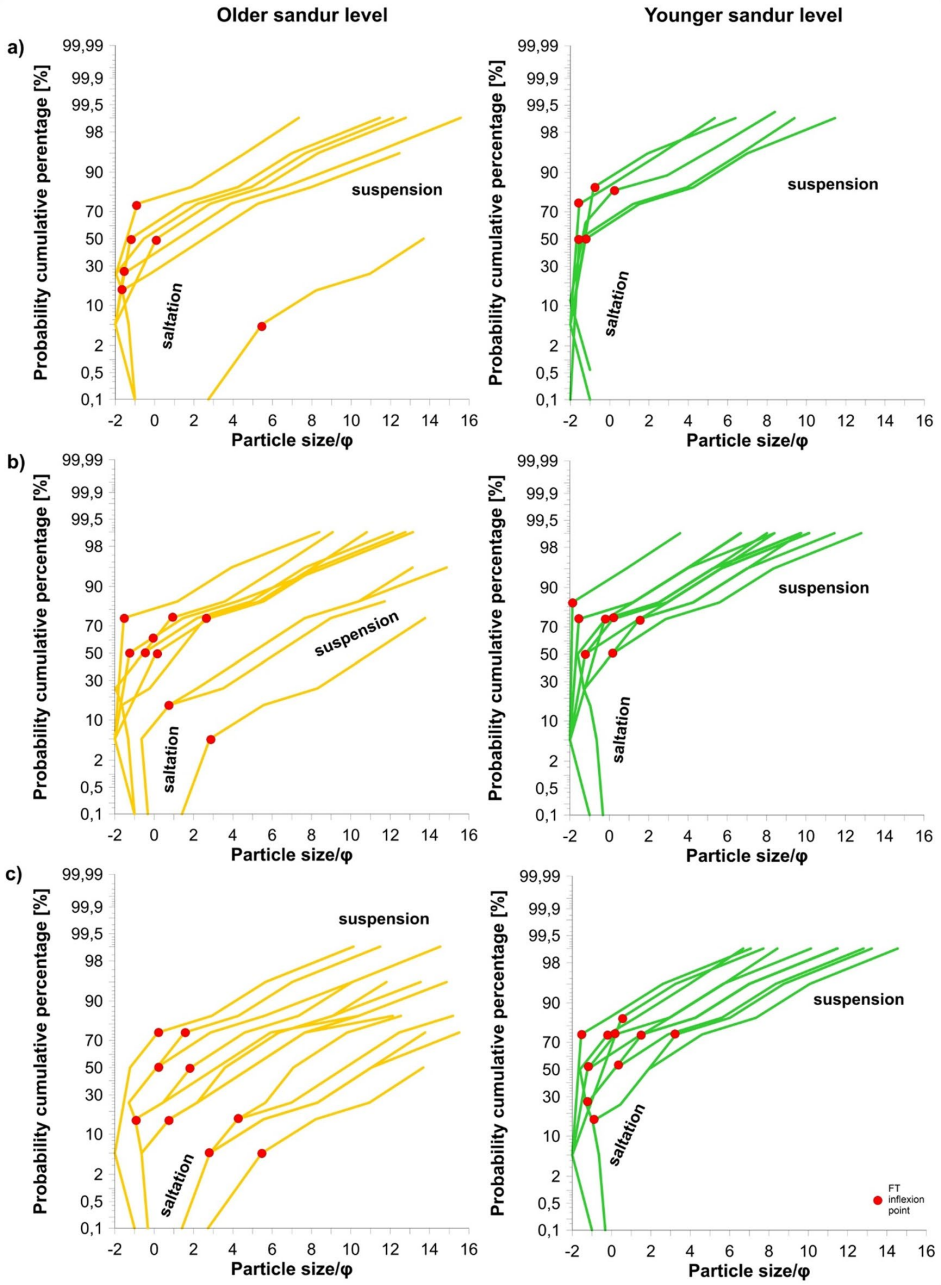
Probability cumulative curves for mineral particles (in φ units) accumulated by the wind in the kettle holes of Skeiðarársandur at both outwash levels in: (**a**) 2021/2022; (**b**) 2022/2023; (**c**) 2023/2024. FT inflexion point indicates the maximum grain size at which transport in suspension is possible. Graphs generated with Golden Software LLC – Grapher 11^140^.

In the context of the presented wind conditions, the cumulative grain size curves show that particle transport was dominated by saltation and suspension at both outwash levels (Fig. 8). The analysis did not identify any samples involving traction transport. In the older sandur level, saltation included about 50% (5–75%) of grains, on average, in the first and second seasons, slightly less than the value quoted by^28^, namely 55–70% of the material. This type of transport is considered the most efficient and usually occurs at a wind speed of 5–10 m s^–1^. 15% (5–75%) of the material in saltation transport was recorded in the third season (**Supplementary Table S2**). The maximum grain size (on average) that could be lifted for suspended transport was granule in the first season (2.00–2.38 mm), coarse sand in the second one (0.84–1.00 mm) and fine sand in the third season (0.125–0.149 mm). It is a larger diameter than is usually dominant in this type of transport, i.e. 0.1–0.15 mm (fine-grain sand)^28^. In the case of the younger sandur level, saltation transport included 63% (50–84%) of grains in samples in the first season, 75% (50–84%) in the second, and, on average, 74% (17–84%) in the third season. The maximum grain size that could be lifted in suspension was very coarse and coarse sand (0.84–2.00 mm).

Considering the topography of the whole area (cf.^123^—**Zenodo_Fig7_Wind-effect-index.png** and ^123^—**Zenodo_Fig8_Wind-effect-index_graph.png**), the older outwash level is most exposed to the aeolian factor in the SE–S–SW wind direction, reaching an average index value of 1.10 to 1.14, while the younger level is most exposed to the aeolian factor in the W–SW–S wind direction. The average wind effect index ranges from 1.04 to 1.09. Wind shadowed conditions at Harðaskriða occur mainly in the NE–N–NW wind direction, with an average index of 0.93–0.95, and at the younger level in the E and NW wind direction (0.96–0.97). Regardless of the wind direction, all kettle holes are in the wind shadow.

### The role of plant colonisation

**Fig. 9:**
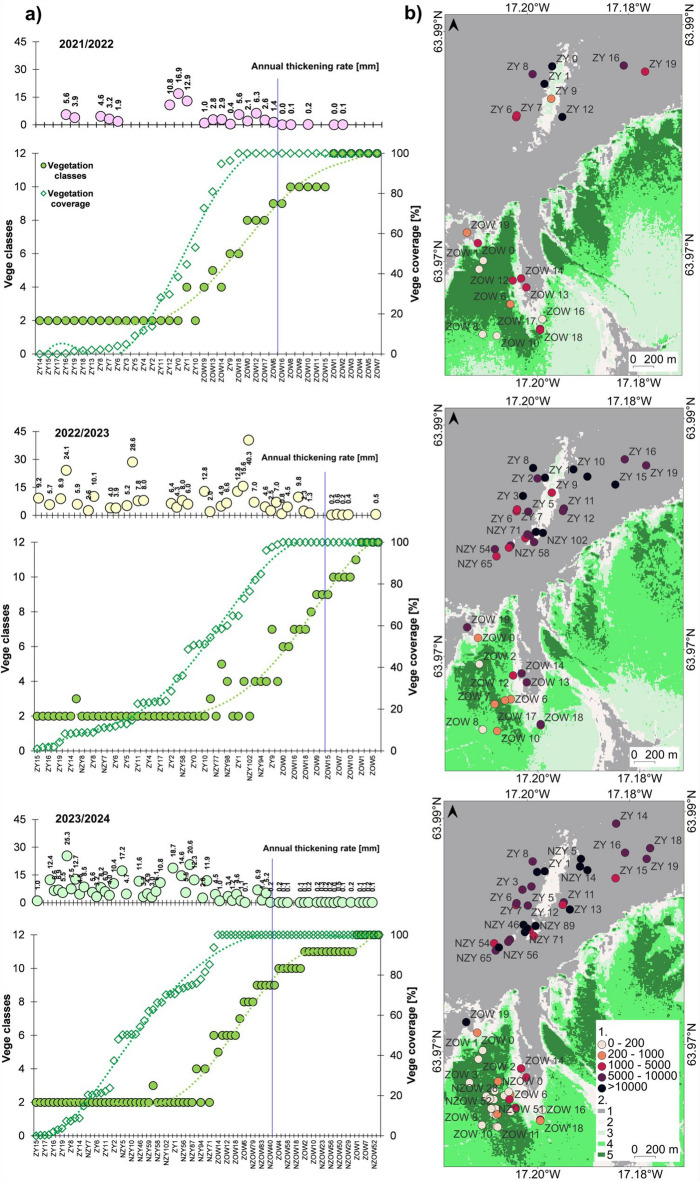
The role of vegetation on the aeolian accumulation within the kettle holes of Skeiðarársandur in 2021/22–2023/24: (**a**) ranking of vegetation classes and vegetation coverage [%] for kettle holes versus annual thickening rate of aeolian sediments [mm yr^–1^], a vertical blue line indicates the border where the thickening rate decreases to 0. Graphs generated with Microsoft Office LTSC Professional Plus 2021 – Excel^124^; (**b**) a spatial distribution of annual accumulation rate within kettle holes on both levels of Skeiðarársandur in the background of vegetation classes calculated from GCI and NDVI: 1—annual accumulation rate [g m^–2^ yr^–1^]; 2—vegetation classes: 1 – lack or sparse vegetation, 2 – low level of vegetation coverage, 3 – medium level, 4 – high level, 5 – kettles entirely covered by vegetation. Maps and image generated with QGIS 3.34.3 Prizren^120^.

Both parameters related to the presence of vegetation on Skeiðarársandur – vegetation classes and vegetation coverage (Fig. 9) (see also ^123^—**Zenodo_aeolian-acc_OlderKH.zip** and ^123^—**Zenodo_aeolian-acc_YoungerKH.zip**) – emphasise its important role for the scale of aeolian accumulation. The degree of vegetation cover clearly distinguishes both outwash horizons, indicating the differentiation of their colonisation time. For Harðaskriða, based on satellite scenes with a ground resolution of 10 m, it is, on average, 97–99%; at the younger horizon, it is, on average, 23.2%, with 50% (IQR) of the values ​​falling within the 8–57% range. The value of GCI and NDVI indices – components of the classification of kettle holes in terms of vegetation cover – depends on the month the satellite scene was acquired, the length of the growing season, and the general meteorological conditions prevailing there. The highest values ​​occurred in the 2021/2022 season, with the largest range of GCI (from −0.07 to 1.79) and NDVI (from −0.04 to 0.45). This season was the mildest in terms of wind and thermal conditions. The sum of positive degree days (PDD) was 2,088 PDD, and the sum of negative degree days (NDD) was −139 NDD. In the next two seasons, it was 2,058 PDD and −276 NDD in 2022/2023, and 1,994 PDD and −195 NDD in 2023/2024, respectively. The range of GCI and NDVI was also lower, from −0.01 to 0.74 and from −0.01 to 0.28 in the second season, and from −0.01 to 0.6 and from −0.01 to 0.22 in the third season. Regardless of the range of index values ​​in a given season, however, the rank abundance curves were characterised by a similar pattern of inflexion points, which enabled a uniform classification of kettle holes by vegetation cover (cf. Figure 9a, ^123^—**Zenodo_GCI_NDVI_Vege-classes.xls** and ^123^—**Zenodo_Table2_Vege-classification.pdf**). For the high level of vegetation coverage and last classes, where the depressions are entirely overgrown (ranks 10–12), the annual thickening rate drops almost to 0, and organic parts dominate the accumulated material. As for the spatial pattern of the vegetation coverage—accumulation rate relationship (cf. Figure 9b), here, too, a relationship is visible that the lower the vegetation coverage, the higher the annual rate of aeolian accumulation. For all three seasons, the average values ​​of thickening rate (linear function, R^2^ = 0.74) and average accumulation rate (exponential function, R^2^ = 0.77) were compared for each vegetation cover class for kettle holes (^123^—**Zenodo_Fig9_Acc-vs-plants.png**). In the lack or sparse vegetation class, the accumulation values ​​at the bottom of the landform are, on average, 5.7–7.2 mm yr^–1^ and 5,360–12,600 g m^–2^ yr^–1^, respectively, in the low degree class – 4.1–4.9 mm yr^–1^ and 2,270–3,490 g m^–2^ yr^–1^, in the medium degree class – 2.6–3.4 mm yr^–1^ and 960–1,480 g m^–2^ yr^–1^, in the high degree class – 1.0–1.8 mm yr^–1^ and 410–630 g m^–2^ yr^–1^, in the class with kettles entirely covered by vegetation – 0–0.3 mm yr^–1^ and 175–270 g m^–2^ yr^–1^ (cf.^123^—**Zenodo_Table2_Vege-classification.pdf**).

### Morphometry of kettle holes and aeolian accumulation

The kettle holes of Harðaskriða have an average area of ​​81.01 m^2^, with 50% of the values ​​falling within the range of 43–154 m^2^ (IQR) (^123^—**Zenodo_Morph_KH_Older.xls**). Their average perimeter is 40.33 m ± 21 m, with 31% of the depressions falling within the 30–40 m range and 83% within the 20–60 m range. Their average maximum depth is 1.38 m, with an IQR between 0.9 and 2.25 m. One-third of the kettle holes are shallow landforms up to 1 m of maximum depth. The average volume of the depressions is 87.15 m^3^, with an IQR between 35 and 264 m^3^. Several extensive and deep landforms in the proximal, northern part of the outwash are a distinct group of depressions associated with melting ice blocks buried in situ, marking the position of the glacier front during the flood^59^. Their volume exceeds 1,000 m^3^ (maximum > 500,000 m^3^), and the maximum depth is usually higher than 10 m (maximum up to almost 30 m).

The younger level kettle holes have an average area of ​​242.44 m^2^ (3 times larger than older forms), of which 50% fall in the range of 136–436 m^2^ (IQR) (^123^—**Zenodo_Morph_KH_Younger.xls**). Their average perimeter is 59.26 m ± 37 m (1.5 times longer than older forms). Most depressions (17%) are concentrated in the 40–50 m range in perimeter, and 75% between 20 and 80 m. Their average maximum depth is 2.45 m (1.8 times deeper than older forms), with an IQR between 1.5 and 4.2 m. The volume of younger depressions is, on average, 285.2 m^3^ (3.3 times capacious than older forms), with an IQR between 100 and 848 m^3^. The maximum reaches a value of almost 31,000 m^3^.

All three seasons differed regarding the relationship of various morphometric parameters with accumulation (Table 1) and on both outwash levels. On the older Skeiðarársandur level, where the kettle holes and their surroundings are covered with compact, diverse vegetation, there is a negative average correlation with the morphometry of the landforms in the first and second seasons; the larger and deeper the landforms, the lower the accumulation value. In the third season, such a relationship was not found. When the wind is stronger and the vegetation season is shorter and colder, the role of vegetation increases to a very high correlation in the second and third seasons. It is probably related to the fact that the largest studied kettle holes are concentrated in the central part of Harðaskriða, i.e. the farthest from plume areas. This area is very densely covered with mosses, vascular plants, including birch trees^52^. The importance of vegetation disappears in warmer and milder seasons regarding wind conditions, such as those in 2021/2022. At the younger level, the situation is different. The morphometry of landforms plays an important role in warmer and weaker wind conditions – a very high positive correlation is noted in the first season; the deeper and larger the landforms, the higher the aeolian accumulation. This relationship weakens in windier seasons – the wind mainly plays a decisive role. Since the vegetation cover is still tiny, and areas with negligible, non-compact vegetation surround the depressions, the plant cover does not play a significant role for now.

**Table 1:** The correlation between annual thickening rate and morphometry parameters of kettle holes of both levels of Skeiðarársandur, and also vegetation indexes in 2021/22–2023/24, statistically significant at minimum 90% of probability (*p* = 0.1 _(a)_; *p* = 0.01 _(b)_; *p* = 0.02 _(c)_; *p* = 0.005 _(d)_; *p* = 0.001 _(e)_; not statistically significant *).

Parameter – older level	2021/2022 (*n* = 11)	2022/2023 (*n* = 11)	2023/2024 (*n* = 28)
Area [m^2^]	–0.53 _(a)_	–0.53 _(a)_	–0.15 *
Perimeter [m]	–0.55 _(a)_	–0.46 *	–0.16 *
Max. depth [m]	–0.43 *	–0.56 _(a)_	–0.25 *
Volume [m^3^]	–0.47 *	–0.57 _(a)_	–0.16 *
GCI	–0.32 *	–0.76 _(d)_	–0.69 _(e)_
NDVI	–0.37 *	–0.75 _(d)_	–0.72 _(e)_
Veg. class	–0.51 _(a)_	–0.71 _(b)_	–0.73 _(e)_

**Parameter – younger level**	**2021/2022** (*n* = 7)	**2022/2023** (*n* = 23)	**2023/2024** (*n* = 28)
Area [m^2^]	0.79 _(c)_	0.13 *	0.48 _(a)_
Perimeter [m]	0.84 _(d)_	0.18 *	0.47 _(a)_
Max. depth [m]	0.85 _(d)_	0.28 *	0.57 _(e)_
Volume [m^3^]	0.74 _(a)_	0.13 *	0.56 _(d)_
GCI	–0.38 *	–0.17 *	0.10 *
NDVI	–0.28 *	–0.20 *	0.09 *
Veg. class	–0.20 *	–0.22 *	–0.13 *

## Discussion

Iceland is located in a specific place, at the junction of the Arctic and Atlantic ocean masses (cf. Figure 1 – an insert map) and on the polar front border^7^. It is reflected in the island’s climate and vegetation zones. The central plateau and the north are the Low Arctic zone, where tundra communities dominate. The remaining areas are the cold-tempered zone, with the dominance of birch habitats (mainly *B. pubescens*). As everywhere in the high latitudes of the Northern Hemisphere, the climate in Iceland has been warming significantly in recent years (e.g.^44–46,142^; cf. Figure 2). It has an impact on the glaciers melting and their recession. Due to the general lowering of the glacier surface, the area of ​​slopes exposed in the higher parts of the marginal zone also constantly expands (cf.^11^). A simultaneous change in the thickness and continuity of permafrost accompanies this process. It is still stable and continuous above 1000 m above sea level, reaching a thickness of about 6 m^143^. However, it thaws already at an 800–900 m altitude above sea level, increasing the risk and number of landslides and avalanches^46^. It is another source of aeolian sediments, important during katabatic winds on glaciers^144^, especially during summer heatwaves^145^. Studies in Antarctica also confirm the increasing importance of dust accumulation from local sources during deglaciation^146,147^.

With such a pronounced deglaciation, there are favourable conditions for the intensification of aeolian processes that support weathering processes (including cryolithogenesis^12–14^). Hence, the deserts of Iceland, lag gravel-, lava- and sandy surfaces^43^, including Skeiðarársandur, are considered to be the most important plume areas of aeolian material of the volcano-fluvial origin^27^. The aeolian deposition is also very high here, exceeding 500 g m^–2^ yr^–1^. The monitoring and data analysis showed that at the current stage of deglaciation of the Skeiðarárjökull forefield in response to climate change, the kettle holes, especially in the younger outwash level, are significant sediment traps for aeolian material. On average, 10–15 times more sediments are retained in them than on flat fragments of the sandur. It confirms the considerable role of strong winds above 5 m s^–1^ and their longevity in the area without vegetation cover^28^. The values ​​of aeolian accumulation within the younger outwash level are comparable to those recorded, for example, on Spitsbergen^28,40,42^, in the Athabasca River Valley in the Canadian Rocky Mountains^37^ or on the Tibetan Plateau in the vicinity of Lake Qinghai^148^, which indicates a local source of aeolian material^3^. Indeed, the depressions studied are entirely located in such a zone (**Supplementary Fig. ****S1**).

The vegetation encroachment inhibits aeolian accumulation almost to zero, as exemplified by the older outwash level, Harðaskriða. It is part of a “vegetation island” up to 3.5 km wide and about 16 km long (**Supplementary Fig. ****S1**). It is surrounded by two extensive plume areas: from the north and north-west by the Gígjukvísl proglacial zone and the Skeiðarárjökull front covered with mineral material, and from the south and south-east by the distal part of Skeiðarársandur with an extensive system of braided rivers, passing into the coastal zone. Nevertheless, with the dominant wind direction from the NE–NNE sectors, its NE–SW extension effectively limits the supply of sediments, which are additionally intercepted by vegetation. The average annual accumulation rate here is similar to or slightly higher than that in areas such as Canada in the Coastal Mountains of British Columbia^35,36^, in the Kangerlussuaq region of Greenland^38,39^ and comparable to the values ​​recorded on the flat parts of the outwash^27^.

The statistical characteristics of grain sizes showed that the aeolian material is local, with a large share of medium and coarse sand particles and even granules (> 2 mm in diameter). At the same time, the material is mostly poorly sorted, and its share in saltation transport is 50–70%. This results from the location of the younger sandur level entirely in the plume areas zone (see **Supplementary Fig. **S1), where vegetation cover is still low. This sandur, whose substrate material is highly diversified due to glacial flood genesis^52^, is subject to intensive cryolithogenesis, providing coarser grains. It is, for example, in contrast to data from Raudasandur (Atlantic coast), where the beach material exposed to much stronger winds (cf.^123^—**Zenodo_Fig1_Meteo_graphs.png**) in northwestern Iceland is finer and well-sorted^33^.

In the case of the older level, mostly covered with compact vegetation, the share of finer fractions and transport in suspension increased, which in the third season was on average 85%. Although there were no wind gusts > 30 m s^–1^ cases, they were long-lasting and more diversified in wind direction (cf. Figure 7c). In this way, Harðaskriða probably received fine material from the glacier front, the Gígjukvísl valley, and the south, from the braided rivers zone. The grain size, the aeolian accumulation rate and transport dynamics data refer here to the values ​​found, for example, in Spitsbergen’s central part, on the Ebbabreen forefield. However, these values ​​differ from those quoted by Wojtanowicz (2010) (^28^—Fig. 6, p. 54) for dusty material on Spitsbergen; only the depressions in the directed N–S cluster in the western part of Harðaskriða show similarity to the dusty data of niveo-eolian origin. Therefore, it should be assumed that they may be related to the accumulation of dust on snow still lying in kettle holes in the winter period, when the strongest wind speeds also occur.

The denser the vegetation, the more compact and further from plume areas, the lower the accumulation. It is reflected in the clear spatial differentiation of the accumulation rate (cf. Figures 4, 5 and 9,^123^—**Zenodo_Table2_Vege-classification.pdf**). It is higher at the younger sandur level, where there is no vegetation or we have a low level of cover with pioneer vegetation such as *Oxyria digyna*, *Rumex acetosella*, *Thymus praecox arcticus*, *Arabidopsis petraea*, bryophytes and grasses (especially *Poa glauca* and *Festuca richardsonii*), which cope well with unstable gravel soil and burial by aeolian sediment^149^. Only the surface of the former englacial esker^150^ after the double-headed embayment in the glacier margin during the 1996 jökulhlaup^103^ shows a slightly higher vegetation cover, although it is still mainly mosses. This place became a bar of large ice block clusters during the flood. The ice-sediment bar forced the bifurcation of the meltwater flow, leading to erosion and leaving the area suspended above the active channels. Subsequent water floods during ablation seasons probably did not reach the esker’s surface, so that plant colonisation could have started earlier. Lower values ​​of aeolian accumulation also characterise the depressions in this part. Due to the NE–SW orientation of the esker and the cluster of depressions, the kettle holes in the cluster follow the course of the dominant wind direction. It is reflected in the power-law relationship between the distance of the depression from the esker origin (where flood waters used to flow out) and the annual aeolian accumulation rate; the greater the distance, the lower the accumulation (see **Supplementary Fig. ****S1****b**).

The complexity between vegetation cover and aeolian accumulation is evident in the older Harðaskriða outwash level. All vegetation cover classes, distinguished based on GCI and NDVI indices, occur here. It is closely related to glacial floods, which, at the end of the LIA, occurred on average once every 10 years^59,113^. No vegetation survives such an element and does not regenerate under repeated high water episodes^52^. Therefore, lush vegetation, diverse in terms of species, indicates that a given fragment of the outwash plain has not experienced flooding for a long time.

The main factors regulating the emergence of plant taxa are their ability to survive under the ice of previous warmer periods (seed bank), currently available seed rain^125^, temperature, sunshine duration, water content of the substrate and various important nutrients, sensitivity to wind, aeolian/volcanic sediment cover and slope stability, sensitivity to sheep grazing, erosion. Jónsdóttir et al. (2005) also indicate homo-/heterogeneity of habitats^7^. For example, the rapid spread of the moss *Racomitrium lanuginosum*, which copes very well in pioneer conditions, on well-drained soils and with higher rainfall, causes the displacement of vascular plants at an early stage of colonisation by unfavourable C/N ratio and low pH.

Later in succession, however, *Racomitrium* will favour the encroachment of other vegetation, especially vascular species. The vegetation will form a species-rich dwarf shrub heath community. Under experimental warming conditions, ^7^ showed that it responds to rising temperatures: the abundance of deciduous and evergreen shrubs increased (450%), the number of bryophytes decreased (18%; e.g. *R. lanuginosum*does not tolerate shading, and also has the lowest regeneration rate^151^) and the tree crown height increased (100%). Such habitats have a chance for better development of soil conditions, and this limits the impact of environmental factors^152^, including strong wind. Dense and diverse vegetation will also be able to capture wind-borne dust and incorporate it into the soil structure, thus fertilizing the habitats. The succession of *Betula pubescens* on Skeiðararsandur began probably in 1998; by then, the trees had reached reproductive capacity^153^. Thanks to the wind shade, many kettles of Harðaskriða are inhabited by single trees or their clusters. The outwash plain is expected to become the most extensive natural birch forest, as it was during the medieval warming^52^. This process is already clearly visible in the oldest, highest parts of Harðaskriða, where kettles are probably related to the jökulhlaup of 1892^66^. As we approach the eastern edge of the higher level, with landforms emerging after the jökulhlaup of 1934 or 1938^66^, we observe an increasing value of the aeolian accumulation, where in the absence of vegetation, it is comparable ​​to the younger level (cf. Figure 9).

Kettle hole morphometry plays a role in the case of seasons with a less significant wind factor, as was the case in 2021/2022. A negative, high and statistically significant correlation was found for landforms of the older Skeiðarársandur level and a positive for the younger one (cf. Table 1). In this way, the role of vegetation is also indirectly reflected – warmer seasons, with more sunshine, less windy, and longer vegetation periods allow for better development of biomass, which has an inhibiting effect on the moved aeolian sediment on vegetated surfaces. In the case of unvegetated areas, the greater the depth of the depression, the more difficult it is to lift sediments from the bottom at lower wind speeds, hence the much higher accumulation, exceeding several times the average values ​​of flat sandur surfaces^43^.

In a broader spatial context, kettle holes as an element of sandur affect the increased diversity of surface features. Topography plays an important role in the dynamics of the aeolian factor^31,154,155^. The formation of the wind shadow allows depressions, especially the deeper ones, to become an archive of the aeolian sedimentation. It is a valuable clue in reconstructing the paleoenvironments in which kettle holes of various origins formed in the European Lowlands. The distinction between aeolian and fluvial sediments is possible thanks to the detailed structure of their layering^22^. In turn, changes in the source areas of aeolian material, characteristic of deglaciation periods^147^, including an increase in the share of sources closer to the deposition area, may be a temporal indicator of environmental changes. The finding of a persistent pattern of transitioning from coarser, unsorted fractions to finer, better-sorted material should be significant here, reflecting the intensity of plant colonisation and longer transport of material, mainly in suspension, due to the deglaciation.

The first years of monitoring allowed me to draw several observations related to methodological issues. The plate collecting samples will be modified using a mesh simulating the natural substrate roughness. It should reduce the problem of underestimating the finest fractions, especially at the older sandur level. In addition, special attention should be paid to the content of organic parts in the samples, which has not been determined for all samples so far. Organic matter affects the bulk density of sediments and constitutes a significant percentage of them at the older sandur level. Continuation and development of the results obtained thanks to the SfM technique will allow for the characterisation of the regularity of aeolian accumulation on a detailed scale (cf. Figure 6), and therefore spatially within the kettle hole and linking its size with the exact vegetation cover and the diversity of species composition. The selection of monitoring sites was appropriate, allowing us to see the spatial diversity of the aeolian accumulation rate. However, additional sites in transition zones that are most interesting from the point of view of the dynamics of aeolian and geomorphological phenomena and in terms of plant succession in response to climate change are planned to be installed.

## Conclusions

Glacial flood-origin kettle holes on sandur constitute significant traps for aeolian material. Due to the wind shadow, the accumulation rate is at least several times higher here than on flat surfaces, especially with no dense vegetation. Although the amount of accumulation is regulated by wind force and the duration of the strong wind series, the spatial pattern of aeolian accumulation is repeated in each season. It is highest in the uncolonised kettles of the younger sandur level after the 1996 jökulhlaup, entirely a part of the plume areas for dust and sand. Thus, the younger outwash horizon represents typical local aeolian transport and accumulation conditions. The presence of coarser grains and poor sorting characterises the material. A significant part of the transport takes place by saltation. With the progressing deglaciation, the source zones of aeolian material will constantly shift behind the retreating glacier. The area of ​​the upper parts of the surrounding slopes, freed from ice due to the glacier surface lowering and the exposure of the rocks to weathering, will also be included in this system. Therefore, the source zone of dusty material should be expected to expand. In the longer term, the disappearance of the ice cover due to climate warming may lead to the disappearance of jökulhlaups and braided rivers in favour of systematic drainage in proglacial valleys^156^.

The encroachment of vegetation, stimulated by climate warming, will stabilise the sand and dust covers and slow down the rate of aeolian accumulation. This rate has dropped to almost zero at the older outwash level, covered with compact vegetation. The accumulated material is finer at this level, especially in its midwestern part and the third season with longer, strong wind series. Although the sorting of the material was still poor, a trend of improvement is visible. The grains also show a tendency towards greater homogeneity in grain size distribution. Most transport takes place through suspension, but still from not very distant sources (< 10 km).

Large, especially deep sandur kettle holes make it difficult to reincorporate accumulated material into aeolian transport, constituting an archive of aeolian accumulation, which, until now, has not been mainly considered as a source of knowledge about aeolian processes of deglaciation periods. Identification of aeolian facies at the bottom of Pleistocene glacial flood-origin sandur kettle holes, their dating and the share of mainly local material in the sediments, the persistent pattern of transitioning from coarser to finer, better sorted particles will inform when vegetation had started entered, and the depression had already been formed, indicating the ice disappearance period.

In recent years, the ecological role of depressions as zones of increased biodiversity and a form of water retention (e.g.^157–160^) known as lentic freshwater systems – LFS^161^ has become increasingly important. It applies to landforms located within the post-Weichselian moraines of the European Lowland. Nevertheless, it should be expected that sandur kettle holes may also fulfil a similar function as in Iceland. An increased number of species concerning the surrounding plains already characterise them. The beginnings of the colonisation of Skeiðdarsandur by *Lupinus nootkatensis* are a cause for concern. It spreads extremely quickly, being the main invasive species. Observations from other locations in Iceland indicate that its presence may cause a significant decline in the biodiversity of these fragile ecosystems^162^, which is also an additional motivation for further comprehensive monitoring of the depressions (including SfM-derived material) and their sensitivity to changing factors.

## Electronic supplementary material

Below is the link to the electronic supplementary material.

Supplementary Material 1

## Data Availability

The data describing the studied kettle holes of both levels of the Skeidararsandur outwash plain in S Iceland, aeolian accumulation characteristics, and main meteorological parameters, including wind conditions and vegetation coverage, have been placed in the international open data repository Zenodo^123^: 10.5281/zenodo.14852374. Other details are available in the article and as **Supplementary Material**.
