# Supplementary material for: Aeolian accumulation rate within the kettle holes on Skeiðarársandur (S Iceland) under climate warming conditions
Source: Sci Rep. 2025 Jul 2;15:22622. doi: 10.1038/s41598-025-07346-2 (PMC12217208; doi:10.1038/s41598-025-07346-2)
Supplement: Supplementary file 1 — Supplementary Material 1 [file 41598_2025_7346_MOESM1_ESM.pdf]

Aeolian Accumulation Rate within the Kettle Holes on Skeiðarársandur (S Iceland) under Climate Warming Conditions

Joanna Ewa Szafraniec[1,*]

[1] University of Silesia in Katowice, Faculty of Natural Sciences, Institute of Earth Sciences, Sosnowiec, 41-200, Poland.
[*] Corresponding author: joanna.szafraniec@us.edu.pl

Supplementary Table S1

No.	2021/2022					2022/2023					2023/2024				
	MZ ϕ (mm)	SC	Sorting class	SK_G	K_G	MZ ϕ (mm)	SC	Sorting class	SK_G	K_G	MZ ϕ (mm)	SC	Sorting class	SK_G	K_G
**YOUNGER LEVEL**															
ZY16	1.28 (0.35–0.42) m.s.	1.33	poorly sorted	−0.21 n.s.	0.88 platyk.	0.70 (0.59–0.71) c.s.	1.23	poorly sorted	1.00 s.p.s.	0.72 platyk.	1.28 (0.35–0.42) m.s.	1.33	poorly sorted	−0.21 n.s.	0.88 platyk.
ZY19	1.28 (0.35–0.42) m.s.	1.23	poorly sorted	−0.14 n.s.	0.41 very platyk.	0.70 (0.59–0.71) c.s.	1.33	poorly sorted	0.82 s.p.s	0.88 platyk.	1.28 (0.35–0.42) m.s.	1.23	poorly sorted	−0.14 n.s.	0.95 mesok.
ZY06	1.28 (0.35–0.42) m.s.	1.33	poorly sorted	−0.21 n.s.	0.50 very platyk.	1.28 (0.35–0.42) m.s.	1.23	poorly sorted	−0.14 n.s.	0.41 very platyk.	1.28 (0.35–0.42) m.s.	1.33	poorly sorted	−0.21 n.s.	0.50 very platyk.
ZY07	1.28 (0.35–0.42) m.s.	1.33	poorly sorted	−0.21 n.s.	0.50 very platyk.	1.28 (0.35–0.42) m.s.	1.33	poorly sorted	−0.21 n.s.	0.88 platyk.	1.28 (0.35–0.42) m.s.	1.23	poorly sorted	−0.14 n.s.	0.95 mesok.
ZY01	0.03 (0.84–1.00) c.s.	1.17	poorly sorted	0.54 s.p.s.	0.88 platyk.	0.26 (0.71–0.84) c.s.	1.00	moderately sorted	0.82 s.p.s.	–	0.26 (0.71–0.84) c.s.	1.00	moderately sorted	0.82 s.p.s.	0.88 platyk.
ZY09	0.26 (0.71–0.84) c.s.	1.00	moderately sorted	0.82 s.p.s.	0.88 platyk.	0.70 (0.59–0.71) c.s.	1.33	poorly sorted	0.82 s.p.s.	0.88 platyk.	–	–	–	–	–
ZY15	–	–	–	–	–	1.28	1.33	poorly sorted	−0.21 n.s.	0.88 platyk.	1.28	1.33	poorly sorted	−0.21 n.s.	0.50

						(0.35–0.42) m.s.					(0.35–0.42) m.s.					very platyk.
ZY12	–	–	–	–	–	0.70 (0.59–0.71) c.s.	1.33	poorly sorted	0.82 s.p.s.	0.88 platyk.	1.28 (0.35–0.42) m.s.	1.33	poorly sorted	−0.21 n.s.	0.88 platyk.
ZY10	–	–	–	–	–	0.70 (0.59–0.71) c.s.	1.23	poorly sorted	1.00 s.p.s.	0.72 platyk.	–	–	–	–	–
ZY11	–	–	–	–	–	1.28 (0.35–0.42) m.s.	1.33	poorly sorted	−0.21 n.s.	0.88 platyk.	1.28 (0.35–0.42) m.s.	1.23	poorly sorted	−0.14 n.s.	0.95 mesok.
ZY03	–	–	–	–	–	0.70 (0.59–0.71) c.s.	1.33	poorly sorted	0.82 s.p.s.	0.88 platyk.	1.30 (0.35–0.42) m.s.	1.23	poorly sorted	−0.14 n.s.	0.72 platyk.
ZY08	–	–	–	–	–	0.70 (0.59–0.71) c.s.	1.33	poorly sorted	0.82 s.p.s.	0.88 platyk.	1.28 (0.35–0.42) m.s.	1.23	poorly sorted	−0.14 n.s.	0.95 mesok.
ZY05	–	–	–	–	–	1.28 (0.35–0.42) m.s.	1.33	poorly sorted	−0.21 n.s.	0.50 very platyk.	1.28 (0.35–0.42) m.s.	1.23	poorly sorted	−0.14 n.s.	0.41 very platyk.
ZY02	–	–	–	–	–	0.70 (0.59–0.71) c.s.	1.33	poorly sorted	0.82 s.p.s.	0.88 platyk.	1.28 (0.35–0.42) m.s.	1.23	poorly sorted	−0.14 n.s.	0.41 very platyk.
NZY98	–	–	–	–	–	1.28 (0.35–0.42) m.s.	1.23	poorly sorted	−0.14 n.s.	0.41 very platyk.	1.28 (0.35–0.42) m.s.	1.33	poorly sorted	−0.21 n.s.	0.50 very platyk.
NZY92	–	–	–	–	–	1.28 (0.35–0.42) m.s.	1.23	poorly sorted	−0.14 n.s.	0.72 platyk.	1.28 (0.35–0.42) m.s.	1.23	poorly sorted	−0.14 n.s.	0.41 very platyk.
NZY58	–	–	–	–	–	1.28 (0.35–0.42) m.s.	1.33	poorly sorted	−0.21 n.s.	0.88 platyk.	1.28 (0.35–0.42) m.s.	1.23	poorly sorted	−0.14 n.s.	0.72 platyk.
NZY14	–	–	–	–	–	0.26	0.90	moderately sorted	1.00 s.p.s.	0.72 platyk.	0.84	0.90	moderately sorted	−0.57 s.n.s.	0.72 platyk.

						(0.71–0.84) c.s.					(0.50–0.59) c.s.				
NZY102	–	–	–	–	–	0.26 (0.71–0.84) c.s.	0.90	moderately sorted	1.00 s.p.s.	0.72 platyk.	–	–	–	–	–
NZY54	–	–	–	–	–	0.70 (0.59–0.71) c.s.	1.33	poorly sorted	0.82 s.p.s.	0.50 very platyk.	0.47 (0.71–0.84) c.s.	1.50	poorly sorted	0.64 s.p.s.	0.88 platyk.
NZY89	–	–	–	–	–	0.84 (0.50–0.59) c.s.	0.90	moderately sorted	−0.57 s.n.s.	0.72 platyk.	0.84 (0.50–0.59) c.s.	0.90	moderately sorted	−0.57 s.n.s.	0.72 platyk.
NZY59	–	–	–	–	–	0.70 (0.59–0.71) c.s.	1.33	poorly sorted	0.82 s.p.s.	0.50 very platyk.	1.28 (0.35–0.42) m.s.	1.23	poorly sorted	−0.14 n.s.	0.72 platyk.
NZY87	–	–	–	–	–	0.70 (0.59–0.71) c.s.	1.23	poorly sorted	1.00 s.p.s.	0.72 platyk.	0.26 (0.71–0.84) c.s.	0.90	moderately sorted	1.00 s.p.s.	0.72 platyk.
NZY65	–	–	–	–	–	0.70 (0.59–0.71) c.s.	1.23	poorly sorted	1.00 s.p.s.	0.72 platyk.	1.28 (0.35–0.42) m.s.	1.23	poorly sorted	−0.14 n.s.	0.72 platyk.
NZY71	–	–	–	–	–	0.70 (0.59–0.71) c.s.	1.33	poorly sorted	0.82 s.p.s.	0.88 platyk.	1.28 (0.35–0.42) m.s.	1.23	poorly sorted	−0.14 n.s.	0.72 platyk.
ZY04	–	–	–	–	–	–	–	–	–	–	1.28 (0.35–0.42) m.s.	1.23	poorly sorted	−0.14 n.s.	0.41 very platyk.
NZY46	–	–	–	–	–	–	–	–	–	–	1.30 (0.35–0.42) m.s.	1.23	poorly sorted	−0.14 n.s.	0.95 mesok.
NZY05	–	–	–	–	–	–	–	–	–	–	1.28 (0.35–0.42) m.s.	1.23	poorly sorted	−0.14 n.s.	–
NZY08	–	–	–	–	–	–	–	–	–	–	0.84	0.90	moderately sorted	−0.57 s.n.s.	0.72 platyk.

											(0.50–0.59) c.s.				
ZY13	–	–	–	–	–	–	–	–	–	–	0.84 (0.50–0.59) c.s.	0.90	moderately sorted	–0.57 s.n.s.	0.72 platyk.
NZY56	–	–	–	–	–	–	–	–	–	–	1.28 (0.35–0.42) m.s.	1.33	poorly sorted	–0.21 n.s.	0.88 platyk.
NZY65S	–	–	–	–	–	–	–	–	–	–	1.28 (0.35–0.42) m.s.	1.23	poorly sorted	–0.14 n.s.	0.95 mesok.
ZY18	–	–	–	–	–	–	–	–	–	–	1.28 (0.35–0.42) m.s.	1.23	poorly sorted	–0.14 n.s.	0.41 very platyk.
ZY14	–	–	–	–	–	–	–	–	–	–	1.28 (0.35–0.42) m.s.	1.33	poorly sorted	–0.21 n.s.	0.50 very platyk.
**OLDER LEVEL**															
ZOW12	1.28 (0.35–0.42) m.s.	1.33	poorly sorted	–0.21 n.s.	0.50 very platyk.	1.28 (0.35–0.42) m.s.	1.23	poorly sorted	–0.14 n.s.	0.41 very platyk.	–	–	–	–	–
ZOW13	1.28 (0.35–0.42) m.s.	1.33	poorly sorted	–0.21 n.s.	0.50 very platyk.	1.72 (0.30–0.35) m.s.	1.33	poorly sorted	–1.00 s.n.s.	0.50 very platyk.	2.30 (0.177–0.21) f.s.	1.06	poorly sorted	–0.64 s.n.s.	1.49 leptok.
ZOW18	1.72 (0.30–0.35) m.s.	1.49	poorly sorted	–0.64 s.n.s.	1.49 leptok.	1.28 (0.35–0.42) m.s.	1.23	poorly sorted	–0.14 n.s.	0.41 very platyk.	1.86 (0.25–0.30) m.s.	0.79	moderately sorted	0.43 s.p.s.	0.95 mesok.
ZOW19	1.28 (0.35–0.42) m.s.	1.23	poorly sorted	–0.14 n.s.	0.41 very platyk.	1.28 (0.35–0.42) m.s.	1.23	poorly sorted	–0.14 n.s.	–	1.86 (0.25–0.30) m.s.	0.79	moderately sorted	0.43 s.p.s.	0.95 mesok.
ZOW17	0.70	1.23	moderately well sorted	1.00 s.p.s.	0.72 platyk.	0.70	1.33	poorly sorted	0.82 s.p.s.	0.88 platyk.	1.28	1.33	poorly sorted	–0.21 n.s.	0.88 platyk.

	(0.59–0.71) c.s.					(0.59–0.71) c.s.					(0.35–0.42) m.s.				
ZOW06	1.49 (0.35–0.42) m.s.	1.76	poorly sorted	−0.68 s.n.s.	1.70 very leptok.	2.30 (0.177–0.21) f.s.	1.06	poorly sorted	−0.64 s.n.s.	1.49 leptok.	2.74 (0.149–0.177) f.s.	0.46	well sorted	–	–
ZOW10	2.74 (0.149–0.177) f.s.	0.26	very well sorted	–	–	1.49 (0.35–0.42) m.s.	1.76	poorly sorted	−0.68 s.n.s.	0.60 very platyk.	3.32 (0.088–0.105) v.f.s.	0.90	moderately sorted	0.57 s.p.s.	–
ZOW00	1.72 (0.30–0.35) m.s.	1.33	poorly sorted	−1.00 s.n.s.	0.50 very platyk.	2.30 (0.177–0.21) f.s.	0.79	moderately sorted	−1.00 s.n.s.	0.95 mesok.	2.30 (0.177–0.21) f.s.	1.06	poorly sorted	−0.64 s.n.s.	1.49 very platyk.
ZOW14	1.28 (0.35–0.42) m.s.	1.13	poorly sorted	−0.21 n.s.	0.50 very platyk.	1.28 (0.35–0.42) m.s.	1.23	poorly sorted	−0.14 n.s.	0.72 platyk.	1.28 (0.35–0.42) m.s.	1.23	poorly sorted	−0.14 n.s.	0.95 mesok.
ZOW08	–	–	–	–	–	3.32 (0.088–0.105) v.f.s.	0.90	moderately sorted	0.57 s.p.s.	–	1.86 (0.25–0.30) m.s.	0.79	moderately sorted	1.00 s.p.s.	0.95 mesok.
NZOW23	–	–	–	–	–	2.30 (0.177–0.21) f.s.	1.06	poorly sorted	−0.64 s.n.s.	–	2.74 (0.149–0.177) f.s.	0.46	well sorted	–	–
ZOW02	–	–	–	–	–	1.49 (0.35–0.42) m.s.	1.76	poorly sorted	−0.68 s.n.s.	0.60 very platyk.	3.32 (0.088–0.105) v.f.s.	0.90	moderately sorted	0.57 s.p.s.	–
ZOW07	–	–	–	–	–	1.49 (0.35–0.42) m.s.	1.76	poorly sorted	−0.68 s.n.s.	0.60 very platyk.	2.88 (0.125–0.149) f.s.	1.23	poorly sorted	−0.57 s.n.s.	–
NZOW83	–	–	–	–	–	–	–	–	–	–	1.86 (0.25–0.30) m.s.	0.79	moderately sorted	0.43 s.p.s.	0.95 mesok.
NZOW56	–	–	–	–	–	–	–	–	–	–	3.32 (0.088–0.105) v.f.s.	0.90	moderately sorted	0.57 s.p.s.	–
NZOW00	–	–	–	–	–	–	–	–	–	–	1.28	1.23	poorly sorted	−0.14 n.s.	0.72 platyk.

											(0.35–0.42) m.s.				
NZOW40	–	–	–	–	–	–	–	–	–	–	2.30 (0.177–0.21) f.s.	1.06	poorly sorted	–0.64 s.n.s.	1.49 leptok.
NZOW51	–	–	–	–	–	–	–	–	–	–	2.30 (0.177–0.21) f.s.	0.79	moderately sorted	–0.43 s.n.s.	–
NZOW52	–	–	–	–	–	–	–	–	–	–	3.32 (0.088–0.105) v.f.s.	0.90	moderately sorted	0.57 s.p.s.	–
NZOW16	–	–	–	–	–	–	–	–	–	–	1.28 (0.35–0.42) m.s.	1.49	poorly sorted	0.07 sym.	0.64 very platyk.
ZOW08S	–	–	–	–	–	–	–	–	–	–	3.32 (0.088–0.105) v.f.s.	0.70	moderately sorted	1.00 s.p.s.	0.41 very platyk.
NZOW58	–	–	–	–	–	–	–	–	–	–	3.32 (0.088–0.105) v.f.s.	0.90	moderately sorted	0.57 s.p.s.	–
NZOW28	–	–	–	–	–	–	–	–	–	–	2.30 (0.177–0.21) f.s.	1.93	poorly sorted	–0.28 n.s.	1.49 leptok.
NZOW12	–	–	–	–	–	–	–	–	–	–	3.32 (0.088–0.105) v.f.s.	0.90	moderately sorted	0.57 s.p.s.	–
ZOW01	–	–	–	–	–	–	–	–	–	–	3.32 (0.088–0.105) v.f.s.	0.90	moderately sorted	0.57 s.p.s.	–
NZOW87	–	–	–	–	–	–	–	–	–	–	2.30 (0.177–0.21) f.s.	1.06	poorly sorted	–0.64 s.n.s.	1.49 leptok.
ZOW11	–	–	–	–	–	–	–	–	–	–	3.32	0.90	moderately sorted	0.57 s.p.s.	0.72 platyk.

											(0.088–0.105) v.f.s.				
**NZOW57**	–	–	–	–	–	–	–	–	–	–	2.74 (0.149–0.177) f.s.	0.26	very well sorted	–	–
**NZOW29**	–	–	–	–	–	–	–	–	–	–	1.86 (0.25–0.30) m.s.	1.06	poorly sorted	0.64 s.p.s.	1.49 leptok.
**ZOW16**	–	–	–	–	–	–	–	–	–	–	2.88 (0.125–0.149) f.s.	1.49	poorly sorted	–0.07 sym.	1.49 leptok.

Supplementary Table S1. Main location, dispersion and shape statistics describing aeolian grains collected in Skeiðarársandur kettle holes (S Iceland) in 2021/2022–2023/2024: MZ – mean particle size in φ units and in mm (v.f.s. – very fine sand, f.s. – fine sand, m.s. – medium sand, c.s. – coarse sand), SC – sorting coefficient, SK_G – skewness (s.p.s. – strongly positive skewed, p.s. – positive skewed, sym. – near symmetrical, n.s. – negative skewed, s.n.s. – strongly negative skewed) and K_G – kurtosis (very platyk. – very platykurtic, platyk. – platykurtic, mesok. – mesokurtic, leptok. – leptokurtic, very leptok. – very leptokurtic).

Supplementary Table S2

No.	2021/2022			2022/2023			2023/2024		
	Percentage of grains [%]		Grain size [ϕ] (mm)	Percentage of grains [%]		Grain size [ϕ] (mm)	Percentage of grains [%]		Grain size [ϕ] (mm)
	Saltation	Suspension	FT	Saltation	Suspension	FT	Saltation	Suspension	FT
**YOUNGER LEVEL**									
ZY16	82	18	0.15 (0.84–1.00) coarse sand	75	25	−0.2 (1.00–1.19) very coarse sand	75	25	0.2 (0.84–1.00) coarse sand
ZY19	50	50	−1.6 (2.83–3.36) granule	75	25	−1.6 (2.83–3.36) granule	17	83	−0.85 (1.68–2.00) very coarse sand
ZY06	50	50	−1.2 (2.00–2.38) granule	50	50	0.2 (0.84–1.00) coarse sand	74	26	1.5 (0.35) medium sand
ZY07	50	50	−1.2 (2.00–2.38) granule	76	24	0.25 (0.84) coarse sand	17	83	−0.85 (1.68–2.00) very coarse sand
ZY01	84	16	−0.75 (1.68) very coarse sand	84	16	−1.85 (3.36–4.00) granule	75	25	−1.5 (2.83) granule
ZY09	76	24	−1.6 (2.83–3.36) granule	75	25	−1.6 (2.83–3.36) granule	–	–	–
ZY15	–	–	–	75	25	0.25 (0.84) coarse sand	74	26	1.5 (0.35) medium sand
ZY12	–	–	–	75	25	−1.6 (2.83–3.36) granule	75	25	0.2 (0.84–1.00) coarse sand
ZY10	–	–	–	75	25	−0.25 (1.19) very coarse sand	–	–	–
ZY11	–	–	–	76	24	0.25 (0.84) coarse sand	17	83	−0.85 (1.68–2.00) very coarse sand
ZY03	–	–	–	75	25	−1.5 (2.83) granule	75	25	1.5 (0.35) medium sand
ZY08	–	–	–	75	25	−1.5	17	83	−0.85

						(2.83) granule			(1.68–2.00) very coarse sand
ZY05	–	–	–	74	26	1.6 (0.30–0.35) medium sand	26	74	−1.2 (2.00–2.38) granule
ZY02	–	–	–	75	25	−1.6 (2.83–3.36) granule	26	74	−1.2 (2.00–2.38) granule
NZY98	–	–	–	50	50	0.2 (0.84–1.00) coarse sand	75	25	1.5 (0.35) medium sand
NZY92	–	–	–	74	26	1.6 (0.30–0.35) medium sand	26	74	−1.25 (2.38) granule
NZY58	–	–	–	76	24	0.25 (0.84) coarse sand	74	26	1.5 (0.35) medium sand
NZY14	–	–	–	75	25	−0.2 (1.00–1.19) very coarse sand	75	25	1.5 (0.35) medium sand
NZY102	–	–	–	75	25	−0.2 (1.00–1.19) very coarse sand	–	–	–
NZY54	–	–	–	75	25	−0.2 (1.00–1.19) very coarse sand	84	16	0.6 (0.59–0.71) coarse sand
NZY89	–	–	–	74	26	1.55 (0.30–0.35) medium sand	74	26	1.5 (0.35) medium sand
NZY59	–	–	–	75	25	−0.2 (1.00–1.19) very coarse sand	74	26	1.5 (0.35) medium sand
NZY87	–	–	–	75	25	−0.2 (1.00–1.19) very coarse sand	75	25	−0.2 (1.00–1.19) very coarse sand
NZY65	–	–	–	75	25	−0.2 (1.00–1.19) very coarse sand	74	26	1.5 (0.35) medium sand
NZY71	–	–	–	75	25	−1.6 (2.83–3.36) granule	75	25	1.5 (0.35) medium sand
ZY04	–	–	–	–	–	–	26	74	−1.25 (2.38) granule
NZY46	–	–	–	–	–	–	17	83	−0.85

									(1.68–2.00) very coarse sand
NZY05	–	–	–	–	–	–	75	25	3.25 (0.105) very fine sand
NZY08	–	–	–	–	–	–	75	25	1.5 (0.35) medium sand
ZY13	–	–	–	–	–	–	75	25	1.5 (0.35) medium sand
NZY56	–	–	–	–	–	–	75	25	0.2 (0.84–1.00) coarse sand
NZY65S	–	–	–	–	–	–	17	83	–0.85 (1.68–2.00) very coarse sand
ZY18	–	–	–	–	–	–	26	74	–1.25 (2.38) granule
ZY14	–	–	–	–	–	–	74	26	1.5 (0.35) medium sand
**OLDER LEVEL**									
ZOW12	50	50	–1.2 (2.00–2.38) granule	50	50	–0.4 (1.19–1.41) very coarse sand	–	–	–
ZOW13	50	50	–1.2 (2.00–2.38) granule	50	50	0.2 (0.84–1.00) coarse sand	15	85	0.8 (0.50–0.59) coarse sand
ZOW18	16	84	–1.75 (3.36) granule	50	50	–0.4 (1.19–1.41) very coarse sand	15	85	0.8 (0.50–0.59) coarse sand
ZOW19	50	50	–0.6 (1.41–1.68) very coarse sand	75	25	2.7 (0.149–0.177) fine sand	15	85	0.8 (0.50–0.59) coarse sand
ZOW17	74	26	–0.9 (1.68–2.00) very coarse sand	75	25	–1.5 (2.83) granule	75	25	0.25 (0.84) coarse sand
ZOW06	26	74	–1.6 (2.83–3.36) granule	18	82	0.8 (0.50–0.59) coarse sand	5	95	2.85 (0.125–0.149) fine sand

ZOW10	4.5	95.5	5.45 (0.016–0.031) medium silt	50	50	−1.2 (2.00–2.38) granule	5	95	2.85 (0.125–0.149) fine sand
ZOW00	49	51	0.1 (0.84–1.00) coarse sand	18	82	0.8 (0.50–0.59) coarse sand	15	85	0.8 (0.50–0.59) coarse sand
ZOW14	50	50	−1.2 (2.00–2.38) granule	75	25	1.0 (0.50) medium/coarse sand	50	50	1.85 (0.25–0.30) medium sand
ZOW08	–	–	–	5	95	2.9 (0.125–0.149) fine sand	16	84	4.3 (0.044–0.053) coarse silt
NZOW23	–	–	–	18	82	0.8 (0.50–0.59) coarse sand	5	95	2.85 (0.125–0.149) fine sand
ZOW02	–	–	–	50	50	−1.2 (2.00–2.38) granule	5	95	2.85 (0.125–0.149) fine sand
ZOW07	–	–	–	50	50	−1.2 (2.00–2.38) granule	16	84	4.3 (0.044–0.053) coarse silt
NZOW83	–	–	–	–	–	–	15	85	0.8 (0.50–0.59) coarse sand
NZOW56	–	–	–	–	–	–	5	95	2.85 (0.125–0.149) fine sand
NZOW00	–	–	–	–	–	–	75	25	1.6 (0.30–0.35) medium sand
NZOW40	–	–	–	–	–	–	15	85	0.8 (0.50–0.59) coarse sand
NZOW51	–	–	–	–	–	–	16	84	4.3 (0.044–0.053) coarse silt
NZOW52	–	–	–	–	–	–	5	95	2.85 (0.125–0.149) fine sand
NZOW16	–	–	–	–	–	–	50	50	0.25 (0.84) coarse sand
ZOW08S	–	–	–	–	–	–	5	95	5.5 (0.016–0.031)

									medium silt
NZOW58	–	–	–	–	–	–	5	95	2.85<br>(0.125–0.149)<br>fine sand
NZOW28	–	–	–	–	–	–	16	84	–0.9<br>(1.68–2.00)<br>very coarse sand
NZOW12	–	–	–	–	–	–	5	95	2.85<br>(0.125–0.149)<br>fine sand
ZOW01	–	–	–	–	–	–	5	95	2.85<br>(0.125–0.149)<br>fine sand
NZOW87	–	–	–	–	–	–	15	85	0.8<br>(0.50–0.59)<br>coarse sand
ZOW11	–	–	–	–	–	–	5	95	2.85<br>(0.125–0.149)<br>fine sand
NZOW57	–	–	–	–	–	–	5	95	5.5<br>(0.016–0.031)<br>medium silt
NZOW29	–	–	–	–	–	–	15	85	0.8<br>(0.50–0.59)<br>coarse sand
ZOW16	–	–	–	–	–	–	15	85	0.8<br>(0.50–0.59)<br>coarse sand

Supplementary Table S2. Statistical characteristic values of the analysis of grain-size cumulative curves (Visher, 1969) – the sediments of the kettle holes in Skeiðarársandur in the 2021/2022–2023/2024 seasons: FT— grain size when a change of transport condition from saltation to suspension was taken place.

Reference:

Visher, G. S. Grain size distributions and depositional processes. *J. Sediment. Petrol.* **39**(3), 1074–1106. https://doi.org/10.1306/74d71d9d-2b21-11d7-8648000102c1865d (1969).

Supplementary Figure S1

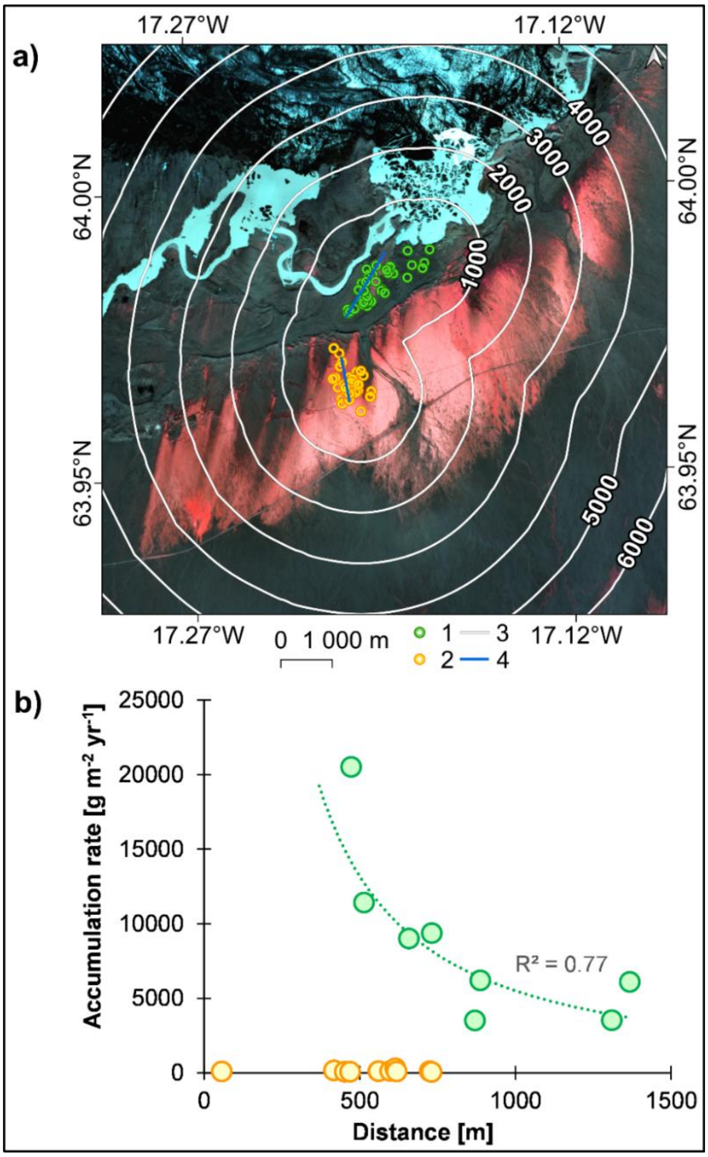

Supplementary Figure S1. Spatial context of the kettle holes location on Skeiðarársandur (S Iceland) within and near the plume areas of the aeolian material: (**a**) distance from the kettle holes zone to the remaining parts of the glacier marginal zone and the axis of the main clusters of oriented depressions where the accumulation rate was monitored in the 2023/2024 season: 1—depressions of the younger outwash plain after the 1996 glacial flood, 2—depressions of the older outwash plain, Harðaskriða, 3—equidistant lines with 1,000 m of interval, 4—axes of oriented depressions on the older and younger outwash plain, the background—Copernicus Sentinel2 data T27WXM_20210809T173316_TCI (B08-B04-B03 composition bands). Retrieved from Copernicus Browser (https://browser.dataspace.copernicus.eu/) on 7 January 2025, processed by the European Space Agency (ESA). Red rasters indicate areas covered with vegetation, blue – ice and water, grey and brown shades – rocks and sediment. Image generated with QGIS 3.34.3 Prizren (http://qgis.osgeo.org); (**b**) relationship between distance from the former subglacial tunnel outlet to kettles along the axis of the oriented clusters on the older and younger levels of the sandur and the aeolian accumulation rate measured in them in the 2023/2024 season ($n = 7$, $p = 0.05$). Graph generated with Microsoft Office LTSC Professional Plus 2021 – Excel (https://www.microsoft.com).

Reference:

QGIS Development Team, QGIS Geographic Information System. Open Source Geospatial Foundation Project. http://qgis.osgeo.org (2025).

Microsoft Corporation. Microsoft Office LTSC Professional Plus 2021 – Excel, wersja 2108. https://www.microsoft.com (2021).
